# Systematic review of quantitative imaging biomarkers for neck and shoulder musculoskeletal disorders

**DOI:** 10.1186/s12891-017-1694-y

**Published:** 2017-09-12

**Authors:** Judith E. Gold, David M. Hallman, Fredrik Hellström, Martin Björklund, Albert G. Crenshaw, Svend Erik Mathiassen, Mary F. Barbe, Sayed Ali

**Affiliations:** 1 Centre for Musculoskeletal Research, Department of Occupational and Public Health Sciences, University of Gävle, Gävle, Sweden; 2 Department of Community Medicine and Rehabilitation, Physiotherapy, Umeå University, Umeå, Sweden; 3 Department of Anatomy and Cell Biology, Temple University Medical School, Philadelphia, PA USA; 4 Department of Radiology, Temple University Medical School, Philadelphia, PA USA; 5 Gold Standard Research Consulting, 830 Montgomery Ave, Bryn Mawr, PA USA

**Keywords:** MRI, MSD, Near-infrared spectroscopy, Pain, Ultrasound

## Abstract

**Background:**

This study systematically summarizes quantitative imaging biomarker research in non-traumatic neck and shoulder musculoskeletal disorders (MSDs). There were two research questions: 1) Are there quantitative imaging biomarkers associated with the presence of neck and shoulder MSDs?, 2) Are there quantitative imaging biomarkers associated with the severity of neck and shoulder MSDs?

**Methods:**

PubMed and SCOPUS were used for the literature search. One hundred and twenty-five studies met primary inclusion criteria. Data were extracted from 49 sufficient quality studies.

**Results:**

Most of the 125 studies were cross-sectional and utilized convenience samples of patients as both cases and controls. Only half controlled for potential confounders via exclusion or in the analysis. Approximately one-third reported response rates. In sufficient quality articles, 82% demonstrated at least one statistically significant association between the MSD(s) and biomarker(s) studied. The literature synthesis suggested that neck muscle size may be decreased in neck pain, and trapezius myalgia and neck/shoulder pain may be associated with reduced vascularity in the trapezius and reduced trapezius oxygen saturation at rest and in response to upper extremity tasks. Reduced vascularity in the supraspinatus tendon may also be a feature in rotator cuff tears. Five of eight studies showed an association between a quantitative imaging marker and MSD severity.

**Conclusions:**

Although research on quantitative imaging biomarkers is still in a nascent stage, some MSD biomarkers were identified. There are limitations in the articles examined, including possible selection bias and inattention to potentially confounding factors. Recommendations for future studies are provided.

**Electronic supplementary material:**

The online version of this article (doi:10.1186/s12891-017-1694-y) contains supplementary material, which is available to authorized users.

## Background

Soft tissue neck and shoulder musculoskeletal disorders (MSDs), namely, disorders of the muscles, tendons, ligaments, nerves, or blood vessels, are prevalent worldwide [1–4], are a common cause of work absence and disability [5], and impose a sizeable societal economic burden [1, 3, 4, 6–11].

Most options for screening, surveillance and diagnosis of proximal upper extremity MSDs depend on symptoms. Improved diagnostic and screening methods, especially objective techniques, are needed [12, 13]. A biomarker has been defined as “a characteristic that is objectively measured and evaluated as an indicator of normal biologic processes, pathogenic processes, or pharmacologic responses to a therapeutic intervention”[14]. Quantitative medical imaging techniques are increasingly used in clinical practice and MSD research, and enable detection of potential MSD biomarkers, including functional and morphological changes. The Quantitative Imaging Biomarkers Alliance and the Terminology Working Group define a quantitative imaging biomarker as “an objective characteristic derived from an in vivo image measured on a ratio or interval scale as an indicator of normal biological processes, pathogenic processes or a response to a therapeutic intervention” [15]. Valid and reliable biomarkers could improve diagnosis and screening methods [16] and provide objective means to evaluate medical treatments and workplace interventions. Use of such biomarkers may also elucidate MSD pathomechanisms.

Three biomarkers classes are conventionally described: exposure, effect (disease), and susceptibility [17]. Herein, we have reviewed biomarkers of effect, defined as “any change that is qualitatively or quantitatively predictive of health impairment or potential impairment…” [17]. Through measurement of biomarkers of effect, pathophysiological processes may be illuminated and used to stage MSD severity, such as early biomarkers that precede disease diagnosis versus late biomarkers in already diagnosed subjects.

### Previous biomarker reviews

Prior reviews on this topic include a pioneering paper highlighting the potential for MSD biomarkers to detect subclinical disease and monitor MSD severity [18], and a later MSD review article [19] focused on biochemical markers. Neither paper mentioned medical imaging. Our recent systematic review also focused on biochemical biomarkers in MSDs [20]. To our knowledge, there have been no published reviews of quantitative imaging biomarkers in neck and shoulder MSDs.

The purpose of this systematic review was to conduct a comprehensive assessment of quantitative imaging biomarkers in neck and shoulder MSDs. We aimed to answer the following two research questions:

1. Are there quantitative imaging biomarkers associated with the presence of neck and shoulder MSDs?
2. Are there quantitative imaging biomarkers associated with the severity of neck and shoulder MSDs?

## Methods

*Review team and process overview.* Our review team consisted of eight researchers with expertise in musculoskeletal radiology and in epidemiologic, intervention and experimental studies, including studies on pathomechanisms within the field of work-related MSD research. The review process was as follows: 1) research questions were formulated; 2) principal concepts of the review were defined; 3) a search strategy and terms were developed (Additional file 1); 4) PubMed and Scopus databases were searched, with results pooled with articles identified from the authors’ files; 5) identified papers were screened based on pre-defined criteria (Additional files 1, 2 and 3) using a two-step procedure of primary (title and abstract) and secondary (quality) screens; 6) summary tables were created from sufficient quality papers; and 7) evidence was synthesized with respect to the two research questions. A consensus process was used throughout the review process. See Gold et al. for further details [20].

*Neck and shoulder MSDs* were defined as clinical diagnoses or musculoskeletal symptoms in the neck and shoulder region. These included both specific and non-specific conditions related to muscles, tendons, nerves, blood vessels or ligaments [21]. The scope of this review encompassed MSDs that occur in a work-related context [22].

*Quantitative imaging biomarker* was defined as an objective characteristic derived from an in vivo image or from an in vivo signal captured in response to electromagnetic radiation to detect morphology or function, measured on a ratio or interval scale as an indicator of normal biological or pathogenic processes [15]. We have focused on minimally invasive/non-invasive methods. Potential quantitative imaging biomarkers could be derived through MRI, ultrasound, far infrared thermography, near infrared spectroscopy (NIRS), laser Doppler flowmetry, and other modalities. Thus, for example, muscle oxygenation as measured through NIRS was included in this review. Because plain radiographs (x-rays) are best utilized in evaluating bone abnormalities and have poor contrast resolution, this imaging modality is not routinely indicated in soft tissue evaluation [23–26]. Thus, studies using only plain radiography were excluded from this review.

*Severity* was operationalized as encompassing longitudinal and cross-sectional differences in symptoms.

*Inclusion criteria.* The current review was limited to studies on adults (age > 18 years) with non-traumatic neck and shoulder MSDs, published between June 4, 1988 and October 14, 2016 and written in English language. Potential biomarkers were examined for the following specific MSDs, as categorized by Boocock, et al. [21]: rotator cuff syndrome/shoulder tendonitis, shoulder capsulitis and thoracic outlet syndrome. Other specific MSDs included are listed in Boocock, et al. [21] Table 2, although status post-whiplash, cervico-brachial fibromyalgia, and joint-related conditions were excluded. Upper extremity non-specific regional pain, namely, “neck pain”, “shoulder pain”, and “neck/shoulder pain”, was also included. We included articles that met our inclusion criteria, even if some parts of the study were consistent with the exclusion criteria; however, only results in compliance with our criteria were included.

*Exclusion criteria* are summarized in Additional file 2.

### Literature search

**Fig. 1:**
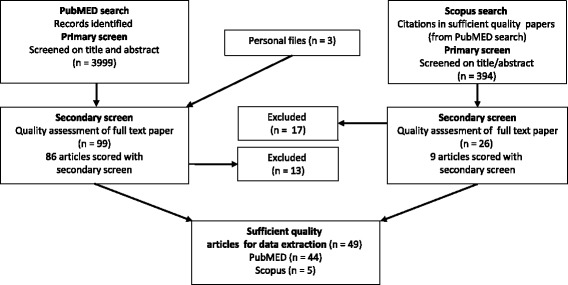
Flowchart of literature search

The search was first conducted in PubMed and combined with articles identified from the authors’ files. Articles were screened for quality. If met, a second search for papers that cited the sufficient quality articles was conducted using Scopus. Additional file 1 provides an overview of the search strategy, while Fig. 1 illustrates the overall search strategy and selection procedure. PubMed search terms included both MESH terms and key words selected for two categories: neck and shoulder MSDs, and biomarkers. Search terms within each category were combined using the “OR” operator, while search terms between categories were combined using “AND”. A systematic procedure was carried out for selection of appropriate MESH terms and key words, where each search term was entered by a step-wise procedure. Fifteen articles were identified by the review team and used for refining the search and testing its sensitivity. See Additional file 4 for the search string.

A total of 4002 articles were examined by members of the review team. The final PubMed search resulted in 3999 articles; three additional papers were added from the team members’ personal files. Primary and secondary screens were implemented (see below and Additional files 1, 2 and 3). The Scopus search identified recently published articles in PubMed that still lacked assigned MESH terms. Since a PubMed search may miss relevant studies in other databases, the Scopus search reduced this potential search strategy bias. To assure clarity and limit reviewer bias, pilot testing of evaluation criteria was conducted at each stage of the review process.

### Primary screen-selection of articles

The primary screen was conducted by two independent reviewers assessing each title and abstract for eligibility based on inclusion and exclusion criteria (Additional file 2), after importing all records from PubMed into systematic review software (EPPI-reviewer4 v4.3.4, EPPI-Centre, Social Science Research Unit, Institute of Education, University of London, UK). The full text was read if necessary. A “yes” answer on any question in Additional file 2 resulted in article exclusion. Results were compared between reviewers, and consensus agreement was reached in all cases (with input from a third person in case of disagreement between reviewers). The same procedure was repeated for articles from personal files and articles found in Scopus.

### Secondary screen-quality assessment and data extraction

All articles passing the primary screen were scored for quality by five review team members. The articles were randomly allocated to five different clusters; each reviewer was assigned randomly to two of these clusters. In the quality screen, each article was assessed by two independent reviewers, scores were compared, and a consensus agreement was reached after discussing disagreements (using a third reviewer as needed). Additional file 3 lists questions used for the quality assessment. These questions were derived from reporting guidelines and checklists for quality assessment in health-related research studies [27–31]. Seventeen items were included in the scoring system, with each item scored as either “yes” (1 point), “unknown or not applicable” (0) or “no” (0). Scores were summed for each paper (range: 0–17). Articles scoring at or above 70% of the maximum (12/17) were labeled as “sufficient quality” and were included for data extraction. Articles scoring “no” on question 15 were excluded from data extraction. These included papers with less than appropriate statistical analysis methods, such as multiple comparisons without adjustment, and modeling without accounting for repeated measures. Data extraction items are listed in Additional file 5.

### Research synthesis

Ulitizing a best evidence synthesis approach, we evaluated the number of sufficient quality articles in order to identify a particular biomarker or class of biomarkers in their potential association with MSD(s) [32]. Considering the biomarker heterogeneity, it was not possible to conduct a meta-analysis. However, it was possible to group results according to the MSD, and then by physiological process or morphology, e.g., hemodynamic and/or oxygenation indicators or muscle dimensions, within diagnoses or symptom designations. An association between a biomarker and an MSD in three or more sufficient quality studies (and at most one sufficient quality study with a null finding) was regarded as evidence that an indicator could serve as a MSD quantitative imaging biomarker. We did not design our review to present different levels of evidence.

## Results

Of the 3999 articles identified through the PubMed (primary) search (Fig. 1), and the three papers added from authors’ files, 99 met secondary screening criteria. Ten papers were excluded after reading the entire article, and three eliminated due to inadequate statistical methods, leaving 86 articles to be scored in the secondary (quality) screen. Forty-four of these met the sufficient quality criteria score. The Scopus database search of these 44 sufficient quality papers yielded 394 citations of which 26 were determined to be non-duplicates and relevant through title and abstract review. Seventeen were eliminated at the secondary screening stage after reading the entire article. Nine articles were scored during the secondary screen, although one was eliminated from data extraction for less than appropriate statistical methods. Of the remaining Scopus identified articles, five scored at ≥12. These five were added to the 44 PubMed identified and similarly scored articles. Thus, 49 studies were regarded to be of sufficient quality for data extraction.

### Secondary screen-quality assessment overview

Additional file 6 shows quality scores of all papers that had undergone secondary screening (*n* = 96; 86 from PubMed and 9 from Scopus). The majority had clearly defined aims, biomarkers, MSDs, and results. All but two unique studies (one longitudinal cohort represented by [33–35] and the other by [36]) had a cross-sectional design, and most utilized convenience samplings of patients, for both cases and referents. Just over half of the studies controlled for confounding factors through exclusion to a particular age or gender, or through a statistical adjustment in the analysis. Thirty-four studies (37%) explicitly stated that those analyzing biomarkers were blinded to case status.

### Data extraction from sufficient quality studies

Additional file 7 gives a descriptive overview of the included studies. The bulk of sufficient quality studies examined neck pain, rotator cuff tears, and trapezius myalgia and other neck/shoulder pain conditions. No sufficient quality studies examined thoracic outlet syndrome. Approximately three-quarters (19/25) of the shoulder disorder studies were conducted in populations with at least one analysis group having an average age of ≥50 years. In contrast, the mean age by analysis groups in the neck pain studies ranged from 22 to 34 years, while the mean age in neck/shoulder studies ranged from 23 to 48 years.

### Are there quantitative imaging biomarkers associated with the presence of neck and shoulder MSDs?

The majority of studies demonstrated an association between at least one biomarker and the MSD(s) examined (Table 1). Only 9/49 (18%) studies reported insignificant findings throughout [37–45].

**Table 1:** Are there quantitative imaging biomarkers associated with the presence of neck and shoulder MSDs?

MSD Classification and diagnosis	Author(s)	Major results (case-control comparison)	Conclusion
Neck disorders and symptoms			
Neck pain	Dibai Filho (2012) [37]	Skin temperature Skin temperature (L & R trapezius), difference btwn sides (thermal asymmetry), NS.	No
Neck pain	Elliott (2008) [46]	Fat index indicating fatty infiltration (relative fat) Fat index: cases < controls, *p* < 0.001 in all muscles.	Yes ↓ fat index in cases in all neck extensor muscles (see Additional file 7).
Neck pain	Falla (2004) [38]	Subcutaneous tissue thickness over SCM, AS SCM subcutaneous tissue thickness (L & R): NS cases vs. controls AS subcutaneous tissue thickness (L & R): NS cases vs. controls	No
Neck pain	Fernández-de-las-Peñas (2008) [47]	Multifidus CSA, muscle shape ratio CSA: ANOVA, group (*p* < 0.001) & cervical level (*p* < 0.001) effects. No interactions. Cases < controls at C3, C4, C5 (*p* < 0.001) & at C6 (*p* < 0.01). Muscle shape ratio: ANOVA, group (*p* < 0.001) & cervical level (*p* < 0.001) effects. Significant interactions btwn group & level (*p* = 0.01). Cases > controls at C3 (*p* < 0.001) & C6 (*p* < 0.01).	Yes ↓ multifidus CSA in cases at C3, C4, C5, C6 ↑ muscle shape ratio in cases at C3, C6
Neck pain	Javanshir (2011) [48]	Lco CSA, anterior-posterior dimension (APD), lateral dimension (LD), and shape ratio (LD/APD) Lco CSA: cases < controls, *p* < 0.001. Lco APD: cases < controls, *p* < 0.01. Lco LD, shape ratio, NS cases vs. controls.	Yes ↓ Lco CSA in cases ↓ Lco APD in cases
Neck pain	Karimi (2016) [53]	Dorsal neck muscle thickness change w. 50% & 100% shoulder MVC in 6 directions Dorsal neck muscle thickness: During MVC: significant interaction of group x muscle, *p* = 0.008. NS, cases vs. controls group x direction; group x force.	Yes Dorsal neck muscle thickness group x muscle effect
Neck pain	Jesus-Moraleida (2011) [49]	Lco thickness, SCM thickness, change of thickness during test/thickness during rest = proportion of muscle recruitment Lco thickness increase throughout all CCFT phases: cases < controls (*p* < 0.001). SCM thickness increase throughout all CCFT phases: NS, cases vs. controls. Lco recruitment: cases < controls, phase 4 (*p* = 0.02), phase 5 (*p* = 0.004), NS other phases. SCM recruitment: NS, cases vs. controls.	Yes ↓ Lco thickness increase throughout all CCFT phases in cases ↓ Lco recruitment, phases 4 & 5
Neck pain	Park (2013) [50]	Mean difference in the bilateral semispinalis capitis muscle thickness Mean difference in the bilateral semispinalis capitis thickness: cases > controls, *p* < 0.05. Within cases mean difference in the bilateral semispinalis capitis thickness: painful side < asymptomatic side, *p* < 0.05.	Yes ↑ mean difference in the bilateral semispinalis capitis thickness in cases ↓ mean difference in the bilateral semispinalis capitis thickness in painful side
Neck pain	Rahnama (2015) [52]	Multifidus muscle thickness change w. shoulder MVC in 6 directions Multifidus muscle thickness: baseline: NS, cases vs. controls; During MVC: significant interaction of group x force, controls > cases (*p* = 0.03). NS, cases vs. controls group x direction; 3- & 4-way interactions involving group.	Yes ↓ multifidus muscle thickness increase in cases during isometric MVC
Neck pain	Sheard (2012) [51]	Differences in water relaxation values (T2 relaxation) quantified from scans before and after exercise were calculated (T2 shift) as a measure of SA muscle activity T2 shift: significant effect for level (*p* = .03) and significant group × level interaction (*p* = .04) but no significant main effect for group (*p* = .59). Post hoc T2 shift: cases > controls at the T6 level (*P* = .02) only.	Yes ↑ T2 shift at T6 in cases
Shoulder disorders and symptoms			
Degenerative rotator cuff lesion	Biberthaler (2003) [54]	Mean functional capillary density, mean capillary diameterMean functional capillary density: lesion < control tissue (*p* < 0.05). Mean capillary diameter: NS, lesion vs. control tissue (*p* > 0.05).	Yes ↓ mean functional capillary density in lesion tissue
Rotator cuff tear (full thickness)	Chang (2014) [56]	Biceps long tendon (BLT) width, thickness, flattening ratio (width/thickness), cross-sectional area, echogenicity ratio BLT width, echogenicity ratio: NS, cases vs. controls BLT thickness: cases > controls, *p* < 0.01. BLT flattening ratio: cases < controls, *p* < 0.01. BLT cross-sectional area: cases > controls, *p* < 0.01.	Yes ↑ BLT thickness in cases ↓ BLT flattening ratio in cases ↑ BLT cross-sectional area in cases
Rotator cuff tear	Choo (2014) [57]	Rotator cable thickness, width Rotator cable thickness: difference among 4 groups (see shoulder tendinosis - Choo), *p* < 0.001; post-hoc analysis – full-thickness tear > normal, *p* < 0.001. Rotator cable width: difference among 4 groups (see shoulder tendinosis - Choo), *p* < 0.001; post-hoc analysis – full-thickness tear > normal, *p* < 0.001; partial-thickness tear > normal^a^.	Yes ↑ rotator cable thickness in full-thickness tears ↑ rotator cable width in full-thickness tears Perhaps ↑ rotator cable width in partial-thickness tears
Rotator cuff tear	Funakoshi (2010) [55]	Vascularity in 4 ROIs: articular & bursal sides of supraspinatus tendon, medial & lateral sides of bursa Non-injected side: cases (RCT) < controls, *p* < 0.0001, in articular & bursal side of the supraspinatus tendon. Injected side: cases (contralateral to RCT) < controls, *p* < 0.0001, in articular & bursal side of the supraspinatus tendon. Cases vs. controls, NS, in medial and lateral side of bursa.	Perhaps ↓ vascularity in articular & bursal sides of supraspinatus in non-injected (rotator cuff tear) side in cases, but may be attributed to age. ↓ vascularity in articular & bursal sides of supraspinatus in injected (rotator cuff intact) side in cases, but may be attributed to age.
Rotator cuff tear	Hirano (2006) [39]	Full vs. partial rotator cuff tear, rotator cuff tear length, amount of subacrominal-subdeltoid bursal fluid Proportion of full & partial tears, NS. Proportion in categorical size of tears, NS. amount of subacrominal-subdeltoid bursal fluid, NS .	No
Rotator cuff tear	Karthikeyan (2015) [58]	Total blood flow in 4 supraspinatus zones, in anteromedial zone, in posteromedial zone Total blood flow in 4 supraspinatus zones: cases (including shoulder impingement – see below) < controls, *p* = 0.001. Anteromedial supraspinatus zone: full-thickness tears < controls, *p* = 0.02; partial-thickness tears vs. controls, NS. Posteromedial supraspinatus zone: full-thickness tears < controls, *p* = 0.04; partial-thickness tears vs. controls, NS.	Yes ↓ supraspinatus blood flow in cases ↓ anteromedial supraspinatus blood flow in full-thickness tears ↓ posteromedial supraspinatus blood flow in full-thickness tears
Rotator cuff tear (full-thickness)	Keener (2015) [35]	Baseline rotator cuff tear width; Width enlargement (defined as ≥ 5 mm compared with that at baseline) percentage Baseline rotator cuff tear width: rotator cuff tear with anterior supraspinatus cable disruption > rotator cuff tear with anterior supraspinatus cable intact, *p* < 0.0001. Width enlargement percentage: NS, rotator cuff tear with anterior supraspinatus cable disruption vs. rotator cuff tear with anterior supraspinatus cable intact .	Yes ↑ baseline rotator cuff tear width with anterior supraspinatus cable disruption.
Rotator cuff tear	Mall (2010) [33]	Rotator cuff tear length, tear width, tear area, rate of substantial tear progression (transformation of a partial-thickness tear into a full-thickness tear or a size increase of > 5 mm in either the width or the length of a full thickness tear compared with that at the time of enrollment) Time of enrollment: full-thickness tear width: symptomatic > asymptomatic, *p* = 0.02; tear length, tear area, NS. Change between visit 1 & visit 2 (see paper for definitions): Shoulder remained asymptomatic: NS, tear length, width, area. Shoulder became symptomatic: tear length: visit 2 > visit 1, *p* = 0.008. tear width: visit 2 > visit 1, *p* = 0.01 tear area: visit 2 > visit 1, *p* = 0.006. Rate of substantial tear progression: symptomatic > asymptomatic, *p* < 0.01	Yes ↑ full-thickness tear width at enrollment in those who later became symptomatic in asymptomatic shoulder. ↑ tear length, width, & area at visit 2 vs. at visit 1 in those who became symptomatic in asymptomatic shoulder. ↑ rate substantial tear progression in in those who became symptomatic in asymptomatic shoulder.
Rotator cuff tear	Moosmayer (2013) [36]	Rotator cuff tear size in anteroposterior plane, in mediolateral plane, tear size increase in anteroposterior plane, in mediolateral plane. Rotator cuff tear size in anteroposterior plane: baseline: NS, symptomatic vs. asymptomatic; 3-year follow-up: symptomatic > asymptomatic, *p* = 0.02 Rotator cuff tear size in mediolateral plane: baseline: NS, symptomatic vs. asymptomatic; 3-year follow-up: NS, symptomatic vs. asymptomatic. Tear size increase in anteroposterior plane: NS, symptomatic vs. asymptomatic. Tear size increase in mediolateral plane: NS, symptomatic vs. asymptomatic.	Yes ↑ rotator cuff tear size in anteroposterior plane at follow-up in tears that became symptomatic
Rotator cuff tear (partial & full) or rotator cuff disease	Keener (2015) [34]	Rotator cuff tear enlargement (see paper for definition) Tear enlargement in 49%; median time to enlargement = 2.8 yrs. tear enlargement: assoc. w. final tear type, *p* < 0.05: full vs. control, HR = 4.17; partial vs. control, HR = 2.73; full vs. partial, HR = 1.53 (all *p* < 0.05, no CI given). New shoulder pain in 46%; median time to pain = 2.6 yrs. shoulder pain assoc. w. final tear type, *p* < 0.05. Assoc. w. tear enlargement, HR = 1.66, *p* < 0.05. 63% became painful before or at tear enlargement; 22% became painful later.	Yes ↑ risk tear enlargement in full-tears vs. controls, in partial tears vs controls, in full-tears vs. partial tears. ↑ risk new shoulder pain w. tear enlargement.
Rotator cuff tear	Terabayashi (2014) [59]	Difference in blood flow peak systolic velocity (PSV), resistance index (RI) between sides Difference between sides in PSV in BA: NS, in any group. Difference between sides in PSV in AHCA: affected > unaffected side in rotator cuff tear with night pain, *p* < 0.001. NS, other groups. Difference between sides in RI in BA: NS, in any group. Difference between sides in RI in AHCA: affected < unaffected side in rotator cuff tear with night pain, *p* < 0.01.	Yes ↑ PSV in AHCA in affected vs unaffected side in rotator cuff tear with night pain. ↓ RI in AHCA in affected vs unaffected side in rotator cuff tear with night pain.
Supraspinatus tendinopathy	Arend (2014) [63]	Maximal supraspinatus tendon thickness (MSTT) MSTT: cases > controls, *p* < 0.05	Yes ↑ MSTT in cases
Rotator cuff tendinitis	Cay (2012) [60]	Subacromial distance, humeral head diameter, Glenoid APD, glenoid articular surface diameter Sagittal subacromial distance: cases < controls, *p* < 0.001 humeral head diameter, glenoid APD, axial glenoid/humerus, and axial glenoid minus humerus, NS in cases vs controls. coronal diameter of humerus: cases < controls, *p* = 0.02. coronal glenoid/humerus, coronal glenoid minus humerus: NS in cases vs controls.	Yes ↓ sagittal subacromial distance in cases ↓ coronal diameter of humerus in cases
Rotator cuff tendinosis	Choo (2014) [57]	Rotator cable thickness, width Rotator cable thickness: difference among 4 groups (see rotator cuff tear - Choo), *p* < 0.001; post-hoc analysis – NS, tendinosis vs controls. Rotator cable width: difference among 4 groups (see rotator cuff tear - Choo), *p* < 0.001; post-hoc analysis – tendinosis > normal, *p* < 0.05^a^.	Perhaps ↑ rotator cable width in tendinosis
Rotator cuff tendinitis	Rechardt (2010) [61]	Carotid artery intima-media thickness Carotid artery imtima-media thickness: NS, in males and females.	No
Shoulder tendinopathy	Joensen (2009) [62]	Supraspinatus tendon thickness Tendon thickness: symptomatic side > asymptomatic side, *p* < 0.01.	Yes ↑ tendon thickness in symptomatic side
Frozen shoulder (Adhesive capsulitis)	Li (2011) [64]	CHL thickness CHL thickness: cases > controls, *p* < 0.001.	Yes ↑ CHL thickness in cases
Frozen shoulder (Adhesive capsulitis)	Michelin (2013) [67]	Joint capsule thickness Joint capsule thickness: cases > controls, *p* < 0.0001	Yes ↑ joint capsule thickness in cases
Frozen shoulder (Adhesive capsulitis)	Song (2011) [65]	Joint capsule thickness in the axillary recess, enhancing portion of the axillary recess thickness, rotator interval thickness Axillary recess: Joint capsule thickness: cases > controls, *p* < 0.001. Axillary recess enhancing portion thickness: cases > controls, *p* < 0.001. Rotator interval Enhancing portion thickness cases > controls, *p* < 0.001.	Yes ↑ axillary recess joint capsule thickness in cases ↑ Axillary recess enhancing portion thickness in cases ↑ Rotator interval Enhancing portion thickness in cases
Frozen shoulder (Adhesive capsulitis	Zhao (2012) [66]	CHL thickness, articular capsule thickness CHL thickness: cases > controls, *p* < 0.001 . articular capsule thickness: cases > controls, *p* < 0.05.	Yes ↑ CHL thickness in cases ↑ articular capsule thickness in cases
Shoulder impingement syndrome	Daghir (2011) [71]	Subacromial-subdeltoid bursal thickness Greatest thickness in any view: NS cases vs. controls. Thickness in shortaxis supraspinatus view: cases > controls, *p* = 0.0009. Thickness in long-axis supraspinatus view: NS cases vs. controls.Thickness in long-axis subscapularis view: NS cases vs. controls.	Yes ↑ subacromial-subdeltoid bursal thickness in cases on shortaxis supraspinatus view
Shoulder impingement syndrome	Hébert (2003) [68]	AHD Cases vs. contralateral control: Flexion: main effect of group, *p* < 0.01, and no interaction with position. Post hoc comparisons: cases < controls at 70, 90, 110 & 130 degrees, *p* < 0.01. Abduction: main effect of group, *p* < 0.01, no interaction with position. Post hoc comparisons: cases < controls at 80, 90, *p* < 0.05 and 110 degrees, *p* < 0.01. Cases vs. contralateral control vs. asymptomatic controls: Flexion - main effect of group, *p* < 0.0001, (position effect, *p* < 0.0001) interaction with position, *p* = 0.01. Post hoc comparisons: cases < asymptomatic controls at 90 & 110 degrees, *p* < 0.01. NS contralateral control vs asymptomatic controls, all positions. Abduction - main effect of group, *p* = 0.052. Post hoc comparisons: cases < asymptomatic controls at 90 & 110 degrees, *p* < 0.01. NS contralateral control vs asymptomatic controls, all positions.	Yes ↓ AHD in cases at 70, 90, 110, 130 degrees flexion vs. contralateral control ↓ AHD in cases at 80, 90, 110 degrees abduction vs. contralateral control ↓ AHD in cases at 90, 110 degrees flexion vs. asymptomatic controls ↓ AHD in cases at in 90, 110 degrees abduction vs. asymptomatic controls
Shoulder impingement syndrome	Karthikeyan (2015) [58]	Total blood flow in 4 supraspinatus zones, in anteromedial zone, in posteromedial zone Total blood flow in 4 supraspinatus zones: cases (including rotator cuff tears – see above) < controls, *p* = 0.001. Anteromedial supraspinatus zone: shoulder impingement < controls, *p* = 0.01. Posteromedial supraspinatus zone: shoulder impingement < controls, *p* = 0.03.	Yes ↓ supraspinatus blood flow in cases ↓ anteromedial supraspinatus blood flow in cases ↓ posteromedial supraspinatus blood flow in cases
Shoulder impingement syndrome	Leong (2012) [69]	AHD, supraspinatus tendon thickness AHD: NS group effect, *p* = 0.08 Supraspinatus tendon thickness: group effect, *p* = 0.002, post-hoc analysis: control volleyball players > controls, *p* < 0.001; cases > controls: *p* = 0.02; NS, control volleyball players vs. cases.	Yes ↑ supraspinatus tendon thickness in cases vs non-volleyball player controls
Shoulder impingement syndrome	Park (2007) [70]	Difference in mean skin temperature btwn sh sides in 5 ROIs Difference in mean skin temperature btwn sh sides anteromedial ROI: cases > controls, *p* = 0.004. anterolateral: cases > controls, *p* = 0.001. posteromedial: cases > controls, *p* = 0.013. posterolateral: cases > controls, *p* = 0.030. lateral: cases > controls, *p* = 0.039.	Yes ↑ difference in mean skin temperature btwn sides in all 5 ROIs in cases
Shoulder pain w. rotator cuff disease (multiple diagnoses)	Kalra (2010) [40]	AHD No group effects at rest (*p* = 0.43) or 45 degrees abduction (*p* = 0.84). No interaction between group and posture.	No
Shoulder pain	O’Sullivan (2012) [41]	Trapezius muscle thickness % change in thickness during contraction vs. rest: NS btwn cases & controls in any of the 4 trapezius regions, at 90 degrees or 120 degrees abduction. Muscle thickness difference between sides at rest or during contractions in cases: NS in any of the 4 trapezius regions, at 0, 90, or 120 degrees abduction.	No
Shoulder pain	Rechardt (2010) [61]	Carotid artery intima-media thickness Carotid imtima-media thickness, NS in males and females. For each standard deviation increase in carotid IMT, risk of unilateral or bilateral sh pain, OR = 1.4 (95% CI 1.0–1.9) for males 60 + .	Perhaps ↑ carotid artery intima-media thickness increases odds of shoulder pain in males 60+
Shoulder pain (internal impingement pain)	Tuite (2007) [72]	Labral length, thick-capsule labrum length, posterior recess angle Labral length: cases > controls, *p* = 0.001. Thick-capsule labrum length: cases > controls, *p* < 0.001. Posterior recess angle: cases > controls, *p* = 0.002. MR arthrogram: greater (dichotomized) glenohumeral internal rotation deficit (GIRD): labral length, thick-capsule labrum length, posterior recess angle, NS.	Yes ↑ labral length in cases ↑ thick capsule labral length in cases ↑ posterior recess angle in cases
Neck/shoulder disorders and symptoms			
Neck/shoulder pain	Hallman (2011) [80]	Muscle blood flow (MBF) During HGT: MBF cases < controls (*p* = 0.02 - ipsi; *p* = 0.04 - contra). After HGT: MBF cases < controls (*p* = 0.001 - ipsi; *p* = 0.003 - contra). During CPT: increase in MBF cases < controls (*p* = 0.04 - ipsi); NS, contra. After CPT: increase in MBF cases < controls (*p* < 0.05 - ipsi); NS, contra.	Yes ↓ MBF in cases during & after HGT in ipsi- and contralateral sides. ↓ increase in MBF during and after CPT in ipsilateral side.
Neck/shoulder pain	Nilsen (2007) [42]	Finger blood flow Finger blood flow: baseline, NS. Response to stressful task: group x time (baseline, 0–10 min, 50–60 min) interaction, *p* = 0.02. Post-hoc comparison: controls vs. cases: *p* = 0.35.	No
Neck/shoulder pain	Shiro (2012) [81]	ΔOHb, ΔHHb, ΔTHb from baseline ΔO2Hb: cases < controls during Relax 3 (*p* < 0.01) & recovery (*p* < 0.05). ΔHHb: NS, cases vs. controls. ΔTHb: cases < controls during Relax 2 & Relax 3 in R trapezius (*p* < 0.05); cases < controls: each Relax & recovery in L trapezius (all *p* < 0.05, except Relax 2 & Relax 3, *p* < 0.001).	Yes ↓ ΔO2Hb in cases during Relax 3 & recovery. ↓ ΔTHb in cases during Relax 2 & Relax 3 in R trapezius; during each Relax & recovery in L trapezius
Neck/shoulder pain	Strøm (2009) [43]	Muscle blood flow At start of work task: cases vs controls, NS difference in blood flow increase in either active or contralateral trapezius. Blood flow during 15 min of recovery in active & contralateral trapezius: cases > controls (*p* = 0.05).	No
Neck/shoulder pain	Takiguchi (2010) [79]	Minimal & maximal standardized uptake values (SUV) of [18F]fluorodeoxyglucose (18F–FDG) Trapezius: mean SUVmax, mean SUVmin: cases < controls, *p* < 0.0001. Presence/absence of neck/shoulder pain and mean SUVmax (R2 = 0.16, *p* < 0.0001), and for SUVmin(R2 = 0.26, *p* < 0.0001), after adjusting for age, gender, smoking status, and diabetes. Gluteus maximus: mean SUVmax, mean SUVmin: NS, cases vs. controls mean. Presence/absence of neck/shoulder pain and mean SUVmax or SUVmin, NS.	Yes
Cervicobrachial pain syndrome	Larsson (1998) [114]	Muscle blood flow Unilateral pain patients: muscle blood flow: painful < asymptomatic side, *p* = 0.01; painful < control, *p* = 0.0009.	Yes ↓ blood flow in painful side in unilateral cases ↓ blood flow in cases
Trapezius myalgia	Acero (1999) [74]	Relative blood volume ANOVA - main effect for group, case < control, during 61–120 s of cold pressor stimulation, *p* = 0.04. All other time points group NS.	Yes ↓ relative blood volume in cases during 61–120 s of cold pressor stimulation.
Trapezius myalgia	Andersen (2010) [44]	ΔOHb, ΔHHb, ΔTHb from baseline ANOVA - main effect of time for all 3ΔxHb (*p* < 0.0001), group x time interaction for OHb (*p* < 0.05). Group effect NS for HHb & THb. Group effect *p*-value for OHb not stated. OHb after exercise increase from baseline: cases < controls, *p* = 0.05.	No
Trapezius myalgia	Cagnie (2012) [75]	Oxygen saturation, muscle blood flow Oxygen saturation: MANOVA - main effects of time, muscle part, and interaction muscle part x group (*p* = 0.049). Post hoc cases < controls in L & R middle trapezius at all time points *p* = 0.03, except 40 min for R middle trapezius (NS). Blood flow: MANOVA - main effects of time, muscle part, and no interaction muscle part x group. No group effect.	Yes ↓ oxygen saturation in L & R trapezius at all but 1 time point.
Trapezius myalgia	Flodgren (2010) [76]	Muscle oxygenation Muscle oxygenation percentage decreased during work (*P* = 0.02), and returned to baseline during recovery.	Perhaps No control subjects were included in this study. Authors conclude normal response in these cases when comparing them to a previous similar study with normal subjects (see Flodgren (2005)).
Trapezius myalgia	Peolsson (2008) [45]	Strain rate, strain rate RMS - before provocation, after provocation, difference after - before NS cases vs. controls: strain rate, strain rate RMS - before provocation, after provocation, difference after - before. After factor analysis with strain rate and strain variables (not velocity variables), followed by clustering, distribution of cases and controls differed, *p* = 0.05. Examination of factors indicated that post-provocation -- most cases have lower levels of strain rate & strain after pain provocation compared with most controls.	No
Trapezius myalgia	Sjøgaard (2010) [77]	ΔOHb, ΔHHb, ΔTHb from baseline Cases: OHb 35 min after start of peg board task < baseline, *p* < 0.05. Controls: OHb not different from baseline. Other OHb, HHb, and THb similar results for cases and controls.	Yes ↓ OHb (vs. baseline) 35 min after start of peg board task in cases, but no change in controls.

^a^: result significant in 1 of 2 radiologists

#### Neck pain (10 studies)

Ten studies examined neck pain [37, 38, 46–53]. Decreased muscle dimensions were observed in cases in the cervical multifidus during rest [47]. In Rahnama et al. [52], no difference in multifidus muscle thickness was observed during baseline, but there was a smaller increase in cases than in controls in muscle thickness from baseline values during isometric maximum voluntary contraction (MVC). An increased muscle shape ratio (ratio between lateral and anterior-posterior dimensions) was seen in the multifidus of cases [47]. Reduced muscle dimensions were also observed in the longus colli [48, 49], and in the semispinalis capitis on cases’ painful side [50]. Dorsal neck muscle thickness change from rest to MVC was different in neck pain cases than in controls, with a tendency toward increased semispinalis capitis thickness in controls, and increased semispinalis cervicis thickness in cases [53]. There was no difference in subcutaneous tissue thickness above the sternocleidomastoid or anterior scalene muscles [38].

Greater serratus anterior muscle activity, measured using fMRI, was observed at thoracic vertebral level 6 in cases [51]. However, less longus colli recruitment, measured by muscle thickness, was found at the greatest flexion angle during incremental nodding [49]. Elliott et al. [46] detected less fat infiltration in neck extensors in neck pain vs. whiplash patients. No difference in trapezius skin temperature, or temperature asymmetry between the left and right trapezii was observed in neck pain vs. controls [37].

#### Rotator cuff tear (11 studies)

Eleven studies investigated rotator cuff tears [33–36, 39, 54–59]. Decreased supraspinatus vascularity or blood flow was observed in two studies [55, 58], and reduced supraspinatus functional capillary density in another [54]. In patients with unilateral rotator cuff tears with night pain, increased peak systolic velocity and decreased resistance index in the anterior humeral circumflex artery was observed in the symptomatic side in comparison to the asymptomatic side [59].

Initially asymptomatic full-thickness rotator cuff tears were examined in two unique longitudinal cohorts. Increased tear dimension and tear progression rate was found in asymptomatic rotator cuff tears that became symptomatic versus those that remained asymptomatic [33]. In this same cohort, Keener et al. [34] found an increased tear enlargement risk in asymptomatic full-thickness tears and in asymptomatic partial-thickness tears versus those with rotator cuff disease, but no tear. In the other longitudinal study, greater rotator cuff tear size was observed in the anteroposterior plane in tears that became symptomatic at 3-year follow-up, although there was no difference in the tear size at baseline [36]. No such increase was observed in the other planes examined. In a cross-sectional study, there was no difference between symptomatic and asymptomatic rotator cuff tears in subacromial-subdeltoid bursal fluid amount, proportion of full- or partial-thickness tears, or tear size [39].

Concommitant to rotator cuff tears, increased dimensions been observed in particular anatomical structures. Greater rotator cable (a fibrous band spanning the insertions of the supraspinatus and infraspinatus) width and thickness were observed in those with full-thickness rotator cuff tears than in healthy subjects [57]. In full-thickness rotator cuff tears, the biceps long tendon (BLT) showed increased thickness and cross-sectional area, and decreased BLT flattening ratio (width/thickness) in comparison to controls [56]. In the first longitudinal study referred to above, greater rotator cuff tear width at baseline was observed in those with anterior supraspinatus cable disruption vs. those without such disruption [35]. However, no difference in tear width enlargement percentage was observed in a minimum of 2 years later.

#### Rotator cuff tendinitis (5 studies)

Five studies examined rotator cuff tendinitis [57, 60–63]. Decreased subacromial distance and humerus diameter [60] were observed in cases. Joensen et al. [62] found increased supraspinatus tendon thickness in cases’ symptomatic side, while Arend et al. [63] observed a greater maximal supraspinatus tendon thickness in cases. Greater rotator cable width was observed in rotator cuff tendinosis than in healthy subjects [57]. No difference in carotid artery intima-media thickness was seen in rotator cuff tendinitis vs. controls [61].

#### Adhesive Capsulitis (4 studies)

Four studies examined adhesive capsulitis (frozen shoulder) [64–67]. Increased coracohumeral ligament [64, 66], articular capsule [66], and axillary recess joint capsule thicknesses [65, 67] were observed in cases. Increased axillary recess and rotator interval contrast enhancement, along with axillary recess thickening were observed [65].

#### Shoulder impingement syndrome (5 studies)

Five studies investigated shoulder impingement syndrome [58, 68–71]. Increased supraspinatus tendon thickness was observed [69]. Decreased acromiohumeral distance (i.e., subacromial distance) was found in one study [68], but not in another [69]. Park et al. [70] found a difference in mean skin temperature between sides (in unilateral shoulder impingement syndrome). Decreased overall supraspinatus blood flow was observed in cases, with less blood flow in specifically in the medial portions of the supraspinatus [58]. Increased subacromial-subdeltoid bursal thickness was observed in one imaging view in cases [71].

#### Shoulder pain (4 studies)

Four studies examined shoulder pain [40, 41, 61, 72]. As the diagnoses were non-specific, we were unable to place them into one of the other more specific categories. Tuite et al. [72] saw an increased labral length, thick capsule labral length and posterior recess angle in cases. No difference was seen in acromiohumeral distance (AHD) between cases and controls at rest or at 45 degrees abduction [40]. Neither was any difference observed between groups in percent change in trapezius muscle thickness between rest and during muscle contraction with shoulder abduction [41]. Rechardt et al. [61] saw increased carotid artery intima-media thickness in males 60+ with shoulder pain, but not in females or in younger cases.

#### Trapezius myalgia, cervicobrachial syndrome and other neck/shoulder pain (12 studies)

Seven studies examined trapezius myalgia and cervicobrachial syndrome, in which muscle hemodynamics, muscle oxygenation or muscle velocity biomarkers were assessed in the trapezius [44, 45, 73–77]. Decreased muscle blood flow was observed in cases versus controls and on the painful side in unilateral cases [78]. A decrease was found in muscle relative blood volume during cold pressor stimulation [74], and oxygen saturation was reduced at baseline and in response to typing [75]. In response to an upper extremity physical task, decreased oxygenated hemoglobin (compared to baseline) was observed in the trapezius in cases, but not controls [77]. No difference in change in trapezius blood flow, or in oxygenated or deoxygenated hemoglobin in response to ergometer exercise was found [44]. Lastly, there was no change in trapezius strain rate/strain rate RMS, a muscle velocity measure, between cases and controls in response to a provocative upper extremity exercise [45].

Five studies examined neck/shoulder pain [42, 43, 79–81]. Decreased trapezius blood flow was seen during and after hand grip and cold pressor tests [80]. Decreased trapezius oxygenated hemoglobin and relative blood volume was observed in response to isometric trapezius contractions [81], but no difference in trapezius blood flow was found in response to a computer work task [43]. Nilsen et al. [42] saw no decrease in finger blood flow in response to a stressful task. Minimal and maximal standardized uptake values of [18F]fluorodeoxyglucose (18F–FDG), glucose metabolism indicator evaluated by PET/CT were lower in trapezii of cases versus controls, but no difference was observed in the control gluteus maximus, even after adjusting for age, gender, smoking status and diabetes [79].

In summary, a) neck muscle size appeared to be decreased in neck pain, and b) reduced blood flow, relative blood volume and reduced oxygen saturation was observed in the trapezius at rest and in response to upper extremity tasks with myalgia and neck/shoulder pain.

### Are there quantitative imaging biomarkers associated with the severity of neck and shoulder MSDs?

Five of eight studies demonstrated a relationship between MSD severity and quantitative imaging biomarkers (Table 2). Four studies investigated a quantitative imaging biomarker in relation to a severity score or disease stage assessment determined during a physical examination. Neck Disability Index (NDI) was negatively correlated with longus colli cross-sectional area (CSA) and anterior-posterior distance in neck pain [48]. No correlation was observed between NDI and trapezius skin temperature [37] or with fat levels in cervical extensor muscles [46]. In rotator cuff tears or rotator cuff disease, American Shoulder and Elbow Score (ASES) was significantly decreased with advancing tear type, and with incident pain in the asymptomatic shoulder [34]. Simple shoulder test score (SST) was similarly reduced.

**Table 2:** Are there quantitative imaging biomarkers associated with the severity of neck and shoulder MSDs?

MSD Classification or diagnosis Author(s)	Biomarker	Severity measure mean & range if available	Symptom duration (mean & SD if available)	Major results (association between biomarker & disease)	Conclusion
Neck disorders and symptoms					
Neck pain Dibai Filho (2012) [37]	Skin temperature	NDI Cases: 8.33 (SD = 2.65) Controls: 2.27 (SD = 1.27)	Unknown	Correlation NDI and skin temperature in right versus left trapezius; thermal asymmetry: NS.	No
Neck pain Elliott (2008) [46]	Fat index indicating fatty infiltration (relative fat)	NDI Cases: 21.9 (SD = 7.5) Controls: 45.5 (SD = 15.9)	Cases: 33.7 (20.6) mo Controls: 20.3 (9.6) mo	Within groups there was no association between NDI and fat levels; total upper fat, *p* = 0.15; total fat, *p* = 0.94. (No description of total vs upper fat in paper)	No
Neck pain Javanshir (2011) [48]	Longus colli muscle CSA, APD, LD, and LD/APD	NDI 33 (SD = 0.5) VAS pain intensity 5.1 (SD = 0.8)	≥ 3 mo^a^	NDI and CSA: rho = −0.45, *p* = 0.05, dominant side; rho = −0.48, *p* = 0.03, non-dominant side. NDI and APD: rho = −0.49, *p* = 0.03, dominant side; rho = −0.45, *p* = 0.05 non-dominant side. NDI and LD or shape ratio, NS. VAS and CSA, APD, LD or shape ratio, NS.	Yes
Shoulder disorders and symptoms					
Rotator cuff tear (partial & full) or rotator cuff disease Keener (2015) [34]	Rotator cuff tear enlargement (see paper for definition), shoulder pain	ASES score - American Shoulder & Elbow Cases: 98.3 (IRQ = 10) Controls: 100(IQR = 0) Surgeons SST score - simple shoulder test (score normalized to 100) Cases: 91.7 (IQR = 33) Controls: 100(IQR = 0) VAS pain intensityCases & controls: 1.0 (IQR = 0)	Unknown	ASES: smaller w. advancing tear type, *p* < 0.05; median decreased by 31.9 points w. new pain, *p* < 0.05. SST: smaller w. advancing tear type, *p* < 0.05; median decreased by 14.8 points w. new pain, *p* < 0.05. VAS: NS. w. advancing tear type; median increased by 3 points w. new pain, *p* < 0.05.	Yes
Shoulder impingement syndrome Park (2007) [70]	Difference in mean skin temperature btwn shoulder sides	VAS pain intensity 6.6 (5.5–9)	22.6 (SD = 40.4) mo	NS differences btwn normal, hypothermic, and hyperthermic cases for VAS (all cases in this analysis, hypothermic defined as abnormally low temperature in involved side vs uninvolved side, hyperthermic defined as opposite of hypothermic).	No
Neck/shoulder disorders and symptoms					
Neck/shoulder pain Nilsen (2007) [42]	Finger skin blood flow	VAS pain intensity: maximal pain response, shoulder pain response, neck pain response Maximal pain response: Cases: 25 (SD = 20.0) Controls: 15 (SD = 16.1) Shoulder pain response: Cases: 17 (SD = 16.9) Controls: 10 (SD = 12.6) Neck pain response: Cases: 20 (SD = 20.3) Controls: 9 (SD = 11.1)	> 3 mo ^a^	Maximal pain response: correlation w. finger skin blood flow response during first 10 min of the stressful task in cases, (rho = 0.52, *p* = 0.004), NS in controls (rho = 0.06, *p* = 0.71.	Yes
Neck/shoulder pain Strøm (2009) [43]	Blood flow	Complaint severity score: neck pain, shoulder pain, musculoskeletal complaint severity index (MCI; mean of 7 pain items). 0 = no complaint; 12 = severe complaint. VAS during experiment: pain intensity and general tension Median (range) cases: neck pain 3 (1–12), shoulder pain 4 (0–12), MCI 2.5 (0.3–4.1) controls: neck pain 0 (0–6), shoulder pain 0 (0–6), MCI 0.5 (0–2.1) These scores are a composite measure of intensity x duration during the 4 weeks preceding the experiment.	3 subjects (2 women) reported having had shoulder and neck pain for less than 12 months, 13 (7 women) for 1–years, three (all men) for 5–10 years, and five (all women) for more than 10 years.	Cases: correlation between pain VAS and blood flow in active trapezius at end of work task (90 min): rho = 0.47, *p* = 0.03. No correlation at 15 or 45 min into work task, *p* > 0.05. Controls: no correlation at 15, 45, or 90 min into work task, *p* ≥ 0.05. Cases: correlation between pain VAS and blood flow in contralateral trapezius at end of work task (90 min): rho = −0.53, *p* < 0.01. No correlation at 15 or 45 min into work task, *p* > 0.05. Controls: similar direction of results, no rho or *p*-values supplied.	Yes
Neck/shoulder pain Takiguchi (2010) [79]	Minimal & maximal standardized uptake values (SUV) of [18F]fluorodeoxyglucose (18F–FDG)	VAS	> 6 mo, with pain at least 1×/mo^a^	Trapezius: mean SUVs & pain VAS (SUVmax: *r* = −0.603, *p* < 0.0001; SUVmin: *r* = −0.405, *p* < 0.0001). Gluteus maximus: mean SUVs & pain VAS, NS.	Yes

^a^: inclusion criteria

Six of eight studies solicited pain ratings from participants through a VAS or other means. Three neck/shoulder pain studies investigated pain severity. In Takiguchi et al. [79], minimal and maximal standardized 18F–FDG uptake values, a glucose metabolism measure, were negatively correlated with VAS pain. In one study, maximal pain response was correlated with finger skin blood flow during the first 10 min of a mentally stressful task in cases, but not controls [42]. In another study, in cases (but not controls) pain and blood flow was positively correlated in the active trapezius, and negatively correlated in the contralateral trapezius at the end of a 90 min computer task [43]. No association was observed between pain rating and longus colli CSA, anterior-posterior distance, or other quantitative imaging parameters examined [48]. Neither was VAS pain related to mean skin temperature differences in shoulder impingement syndrome [70].

In summary, very few studies reviewed found associations between quantitative imaging biomarkers and neck and shoulder MSD severity. As might be inferred from results to our first research question, functional impairment in neck pain may be associated with reduced longus colli dimensions. Functional impairment in rotator cuff disease in an asymptomatic shoulder may be correlated with increasing tear type and incident shoulder pain. In neck/shoulder syndromes, increased pain may be associated with reduced glucose metabolism and increased blood flow in the active trapezius in response to a computer task.

## Discussion

In this study we have summarized the current state of quantitative medical imaging marker research in neck and shoulder MSDs by conducting a comprehensive systematic review. A critical approach was used to synthesize results for the two research questions: 1) are there quantitative medical imaging markers associated with the presence of neck and shoulder MSDs, and 2) are there quantitative medical imaging markers associated with the severity of neck and shoulder MSDs? Within the studies of sufficient quality, we found associations between quantitative medical imaging biomarkers and neck and shoulder MSDs, and were able to identify several commonalities.

Evidence was found for the following quantitative imaging biomarkers: With respect to referents, decreased neck muscle size was observed in cases with neck pain [47–50, 52]. Reduced trapezius blood flow and relative blood volume [73, 74, 80, 81] and oxygen saturation at rest and in response to upper extremity tasks [75, 77, 81] occurred with trapezius myalgia and neck/shoulder pain. Lastly, reduced blood flow and altered vascular parameters were observed in rotator cuff tears [54, 55, 58, 59].

In contrast to the first research question, associations between biomarkers and the severity of neck and shoulder MSDs were observed in only a few studies. Most notably, minimal and maximal standardized 18F–FDG uptake values, a biomarker of trapezius metabolism in neck/shoulder pain, were inversely correlated with pain, indicating reduced muscle metabolism in this condition. However, this was found in only one study [79]. The small sample size resulting in reduced ranges of severity measures in many studies examining neck and shoulder MSD severity may have hampered the feasibility of detecting statistically significant results for our second research question.

A possible explanation for the decreased size of deep neck muscles in neck pain cases advanced by several articles [47–50] is the development of muscle atrophy due to a long term reduction in muscle activity through either pain or reflex inhibition. This explanation is consistent with a smaller change in multifidus muscle thickness during MVC from rest in those with neck pain in comparison to control subjects [52]. In another study, cases and controls showed different patterns of muscle thickness alterations during MVC when compared with rest [53]. A possible mechanism for activity changes during muscle pain is the redistribution of activity from painful muscles or painful areas to adjacent or synergistic muscles, as described in the pain adaptation model [82]. Subjects with neck pain showed reduction in deep neck muscle activity in the longus colli [49]. This was corroborated by prior studies showing reduced strength and endurance during neck flexion tests in subject with neck pain [83, 84]. However, causal relationships cannot be deduced due to the cross-sectional design of sufficient quality studies.

The pathophysiology associated with reduced blood flow, relative blood volume and oxygen saturation with trapezius myalgia and neck/shoulder pain is not clear. Decreased oxygenation as presented by several studies [75, 77] may be related to a reduction in oxygen delivery to the muscle or to increased muscle oxygen consumption. Previous studies using muscle microdialysis found increased pyruvate and lactate, metabolites related to increased anaerobic energy production, in painful trapezius muscles [77, 85]. Findings of reduced trapezius muscle blood flow in response to physical load [73, 80, 81] or pain induced during an experiment [74, 80] does not oppose the hypothesis of reduced oxygen delivery. Reduced blood flow may be attributed to an imbalance between vasoconstriction and dilatation in muscle arterioles [86]. This imbalance could be due to aberrant activation in the sympathetic nervous system or downregulation of adrenoreceptors in the arteriole epithelium in patients with MSDs [87]. Indeed, in some sufficient quality studies in this review, patients with MSDs show aberrant sympathetic activity compared to asymptomatic controls [75, 77, 80], although adrenoreceptor expression was not investigated. Together, the reduction in blood flow and oxygen saturation may facilitate the production of muscle metabolites like lactate, which are known to influence muscle nociceptor activity.

### Limitations of the review

Other techniques besides imaging are available for measuring some of the functional and morphological features or processes addressed in this review. However, these other methods for assessing biomarkers were beyond the scope of the present review. For instance, our inclusion criteria allowed for articles on photoplethysmography to measure blood pressure, but not strain-gauge plethysmography. Although the term “plethysmography” was part of our search string, no papers were found that utilized strain-gauge plethysmography in neck or shoulder MSDs. Studies using plain x-rays to the exclusion of other imaging modalities were excluded. Plain radiographs are best utilized in evaluating osteoarthritis, fractures, dislocations and other bone abnormalities, and are not routinely indicated in soft tissue MSDs [23–26]. However, we may have missed some biomarkers that could be of interest such as calcifications, soft tissue swelling, or acromial abnormalities including variant acromial morphology and acromial spurs.

### Methodological limitations in the articles reviewed

#### Selection bias - response rate

The response rate to participate could be ascertained in only 15 (31%) of the 49 sufficient quality studies. Without response rates, selection bias cannot be adequately assessed. Hence, it is unknown if the cases and controls represent the underlying population, or to what extent they may be comparable. We recommend including response rates for both cases and controls in future quantitative imaging studies.

#### Confounding

Approximately half of the reviewed sufficient quality studies controlled for potential confounders, either through restriction of study subjects (e.g., by age or gender) or through adjustment in the statistical analysis. With respect to the quantitative imaging parameters reviewed here, muscle oxygenation, including in the trapezius, was found to be greater in males than females in many studies [88–91]. However, gender had no influence on erector spinae oxygenation in a sustained trunk extension test [92]. This latter study also found no difference in relative blood volume with respect to gender. But, literature is sparse in this area. Muscle oxygenation and blood volume responses in limb muscles are significantly influenced by both age [93] and level of exercise training [94], yet no study has looked at the effects on shoulder and neck muscles.

Trapezius muscle size is greater in males than females [91]. In a biopsy study, Lindman et al. [95] found that female trapezius muscle fibers have smaller cross-sectional areas than males, and more type II fibers. Neck muscle size may also differ by gender. Zheng et al. [96] found a greater total neck muscle volume in males versus females. However, the proportion of each muscle volume examined in comparison to total neck muscle volume was similar between genders, except for the sternocleidomastoid, longus capitis, and obliqus capitis inferior. Deep neck posterior muscles and semispinalis capitis cross-sectional areas were larger in males than females, but not after adjusting for body weight [97]. In that study, muscle shape ratio did not differ by gender. Nor were there any differences in muscle dimension by age.

Although several studies suggested that reduced vascularity in the supraspinatus tendon may be associated with rotator cuff tears, two of these studies used much younger controls than cases [55, 58] (see Additional file 7). Due to the design of these studies, it is difficult to determine whether the results were due to age or to pathology. Rudzki et al. [98] found reduced blood flow in the supraspinatus tendon in those over 40 years in their study of asymptomatic rotator cuff tears, which roughly corresponds to the differentiating age between the two groups in the above studies.

The above findings suggest that (minimally) age, gender, exercise frequency, and BMI should be collected from study subjects and controlled for, either during analysis or through selection.

#### Directions for future research

Despite the similarity of symptoms in neck and neck/shoulder disorders, there is a marked difference in the imaging metrics obtained in studies with these two symptom designations. Muscle dimensions have been researched in neck pain, but not in trapezius myalgia and other neck/shoulder disorders. Conversely, muscle oxygenation and relative blood volume have been explored in the trapezius, but not in other neck muscles. Future research should examine muscle dimensions in the trapezius, and muscle oxygenation and relative blood volume in other muscles.

The research on muscle dimension, oxygenation and relative blood volume has been conducted in subjects with different MSD labels, i.e., in neck pain, trapezius myalgia and neck/shoulder pain. Here, we used the diagnosis or syndrome name presented in the articles. These diagnoses or syndrome names are based on the painful region. However, the division between neck and shoulder is not clear. For example, when considering functional anatomy, the neck and upper trapezius could be considered as the same region thus rendering definitions of neck and neck/shoulder regions arbitrary. Furthermore, there are suggestions of a possible common pathophysiological mechanism in these syndromes [99].

As mentioned previously, a majority of shoulder studies were conducted in older populations (at least one analysis group with mean age ≥ 50 years), whereas the neck and neck/shoulder studies were conducted in younger populations (mean age < 50 years). This could be due partially to the average age at onset of these disorders. However, given the potential for a possible spectrum effect [100], it would be of interest to study a broader range of ages.

Only 12 of the 49 sufficient quality studies in this review listed the duration of symptoms in patients (range: 9.1–114 months), all of which durations are chronic by definition [36, 38, 40, 41, 46, 47, 64, 66, 68, 70, 73, 76, 80]. One review has determined that blood flow increases to the site of rotator cuff small tears, but that decreased vascularity is observed as tear size increases and the healing response fails [101]. This suggests that varying results in vascularity in the rotator cuff tendons may be influenced by symptom duration. In view of pathophysiological mechanism research, we recommend that quantitative imaging biomarkers be investigated in MSD patients with shorter symptom durations. We further recommend that quantitative imaging biomarker study be report the range of symptoms and their duration.

Although focused on computed tomography imaging methods, animal models would suggest that different quantitative imaging biomarkers and findings are present at different MSD stages [102–105]. In humans, various quantitative imaging biomarkers reflective of underlying musculoskeletal changes are valid at different stages of disease. For instance, the AHD decrease is a late stage phenomenon. It is detectable in large chronic full-thickness rotator cuff tears, but not in earlier stages of rotator cuff disease [106]. The question of which imaging modality best captures the particular biomarker under consideration is beyond the scope of this review. Determining the most appropriate imaging modality for a given quantitative imaging biomarker is an essential area for future research.

#### Heterogeneity – other considerations

Quantitative imaging has the potential to be unbiased and precise, particularly in comparison to ordinal scales such as the Bigliani classification [107] sometimes used in shoulder impingement syndrome. As with all types of biomarkers, optimally, a complete analytical evaluation should be conducted for each quantitative imaging biomarker under consideration. This evaluation should include determination of limit of detection, limit of quantification, reference values in normal subjects, as well as assessing the reliability and validity of any such biomarker [108]. There are unique considerations for quantitative imaging. Sources of variability include the instrument/acquisition system, and the image measurement algorithm, as well as the patient [109]. For instance, patient motion may affect the performance of the imaging acquisition system [110], and image processing software may include a number of steps, each of which requires validation [108]. See Raunig et al. [109] for a thorough review of statistical methods for assessing technical performance in quantitative imaging. These technical considerations must be addressed prior to validating the clinical utility of any suggested quantitative imaging biomarker [108, 109].

### Recommendations

Below are our brief recommendations for future quantitative imaging biomarker research:

1. Report the response rate for all analysis groups.
2. Carefully consider and report potential confounders, gather information on these factors from study subjects, and potentially control for them through exclusion or through adjustment in the statistical analysis.
3. Report symptom duration and/or severity in study subjects.
4. Clearly describe MSD case definition criteria, including a description of localization of symptoms.
5. Prioritize quantitative imaging biomarker studies that are longitudinal.

## Conclusions

Based on our comprehensive review, there is limited evidence of an association between quantitative medical imaging biomarkers and neck and shoulder MSDs. The most consistent studies suggest that deep neck muscle size holds promise as a biomarker for neck pain, and that trapezius blood flow, relative blood volume, and muscle oxygenation are worthy of consideration as biomarkers for trapezius myalgia and neck/shoulder pain. Further research is warranted. In the meantime, clinicians may find value in our findings. For instance, radiologists may wish to adjust imaging scan planes to allow better volumetric analysis, and refine protocols to better characterize blood flow. Some quantitative imaging parameters, e.g., muscle size and blood flow, are not routinely included in radiology reports. It may behoov the clinician to do so. Additionally, epidemiologists may wish to include these biomarkers in cross-sectional and prospective studies of neck and shoulder MSDs. Prospective high quality studies are needed as this discipline moves forward. Future testing should be done with regard to MSD symptom duration and severity. Results should be reported with consideration to the effects of potentially confounding factors (minimally including age, gender, and exercise), and response rates of all analysis groups should be described so that potential selection bias may be assessed.

## Additional files

Additional file 1: Search terms for musculoskeletal disorders (MSDs) and imaging markers. (PDF 14 kb)

Additional file 2: Questions used in the primary screen for exclusion of articles. (PDF 9 kb)

Additional file 3: Questions used in the secondary screen for quality assessment. (PDF 13 kb)

Additional file 4: PubMed search string. (PDF 5 kb)

Additional file 5: Data extraction items. (PDF 9 kb)

Additional file 6: Quality scores for each of the reviewed papers in the primary screen, including the papers of sufficient quality (≥70%) and insufficient quality (< 70%) [114–159]. (DOCX 177 kb)

Additional file 7: Overview with descriptive information of included studies, by anatomical region of the disorder. (DOCX 112 kb)

## Abbreviations

18F–FDG: [18F]fluorodeoxyglucose (Glucose analog where one hydoxylgroup has been replaced with a radioacitve fluorine-18 isotope, an indicator of tissue glucose uptake in PET)
ADP: Anterior-posterior dimension
AHCA: Anterior humeral circumflex artery
AHD: Acromiohumeral distance
ANOVA: Analysis of Variance
AS: Anterior scalene
ASES: American Shoulder & Elbow Score (A validated instrument to assess shoulder pain and function [111])
Assoc.: Associated
BA: Brachial artery
BLT: Biceps long tendon
BMI: Body mass index (A measure of body fat based on height and weight)
C3-C6: Cervical vertebrae
CCFT: Craniocervical flexion test
CHL: Coracohumeral ligament
CI: Confidence interval
CPT: Cold pressor test (A cardiovascular test involving immersion of the dominant hand up to the wrist for 1–3 min in cold water)
CSA: Cross - sectional area
HGT: Static hand grip test (A test in which the subject presses a dynamometer with their dominant hand [80])
HR: Hazard ratio
IQR: Interquartile range
Lco: Longus colli muscle
LD: Lateral dimension
LD/ADP: Muscle shape ratio (ratio between lateral and anterior-posterior dimensions)
MANOVA: Multivariate analysis of variance
MBF: Muscle blood flow
MCI: Musculoskeletal complaint severity index (Mean value of 7 pain areas [43])
MR: Magnetic resonance
MRI: Magnetic Resonance Imaging
MSTT: Maximal supraspinatus tendon thickness
MVC: Maximum voluntary contraction
NDI: Neck Disability Index (A validated instrument to assess disability due to neck pain [112])
NIRS: Near infrared spectroscopy
NS: Not significant
OHb: Oxygenated hemoglobin
OR: Odds ratio
PET/CT: Positron-emission tomography/computerized tomography
PSV: Peak systolic velocity
RCT: Rotator cuff tear
RI: Resistance index (RI = (PSV (see above) – end diastoic velocity)/PSV)
RMS: Root mean square
ROI: Region of interest
SA: Serratus anterior muscle
SCM: Sternocleidomastoid muscle
SD: Standard deviation
SIS: Shoulder impingement syndrome
SST: Simple shoulder test score (An instrument to assess shoulder functional disability [113])
SUV: Standardized uptake values (Measurment for quantification in positron-emission tomography)
T6-T10: Thoracic vertebrae
TVI: Tissue velocity imaging
VAS: Visual Analogue Scale
VO2max: Maximal oxygen uptake
ΔHHb: Change in de-oxygenated hemoglobin (Determined through NIRS, from a baseline value)
ΔOHb: Change in oxygenated hemoglobin (Determined through NIRS, from a baseline value)
ΔTHb: Change in total hemoglobin, interpreted as relative blood volume (Determined through NIRS, from a baseline value)

## References

[1] Hassard J, Teoh K, Cox T, Dewe P, Cosmar M, Grundler R (2014). Calculating the cost of work-related stress and psychosocial risks - a literature review. The European Agency for Safety and Health at work.

[2] Huisstede BM, Bierma-Zeinstra SM, Koes BW, Verhaar JA (2006). Incidence and prevalence of upper-extremity musculoskeletal disorders. A systematic appraisal of the literature. BMC Musculoskelet Disord.

[3] Health and Safety Executive. Cost benefit studies that support tackling musculoskeletal disorders. Research Report 491, 2006. http://www.hse.gov.uk/research/rrpdf/rr491.pdf. Accessed 14 Aug 2017.

[4] World Health Organization. Physical Inactivity: A Global Public Health Problem, http://www.who.int/dietphysicalactivity/factsheet_inactivity/en/. 2014. Accessed 8 Oct 2014.

[5] Schneider E, Irastorza X (2010). OSH in figures: work-related musculoskeletal disorders in the EU — facts and figures.

[6] Buckle PW, Devereux JJ (2002). The nature of work-related neck and upper limb musculoskeletal disorders. Appl Ergon.

[7] National Academy of Sciences (2001). Panel on musculoskeletal disorders and the workplace commission on behavioral and social sciences and education, National Research Council. Musculoskeletal disorders and the workplace: low back and upper extremities.

[8] Virta L, Joranger P, Brox JI, Eriksson R (2012). Costs of shoulder pain and resource use in primary health care: a cost-of-illness study in Sweden. BMC Musculoskelet Disord.

[9] Coyte PC, Asche CV, Croxford R, Chan B (1998). The economic cost of musculoskeletal disorders in Canada. Arthritis Care Res.

[10] Bureau of Labor Statistics (2017). The cost of care: new insights into healthcare spending growth.

[11] OSHA (2014). Prevention of work-related musculoskeletal disorders. U.S. Department of Labor, Occupational Safety & Health Administration.

[12] Viikari-Juntura E (1999). New avenues in research on musculoskeletal disorders. Scand J Work Environ Health.

[13] Punnett L, Gold JE, Johansson H, Djupsjöbacka M, Windhorst U, Passatore M, Ljubisavljevic M, Blair SJ (2003). Work-related upper extremity disorders: epidemiologic findings and unresolved questions. Chronic work-related Myalgia: neuromuscular mechanisms behind work-related chronic muscle pain syndromes.

[14] Institute of Medicine (2010). Evaluation of biomarkers and surrogate endpoints in chronic disease.

[15] Kessler LG, Barnhart HX, Buckler AJ, Choudhury KR, Kondratovich MV, Toledano A (2015). The emerging science of quantitative imaging biomarkers terminology and definitions for scientific studies and regulatory submissions. Stat Methods Med Res.

[16] Mayeux R (2004). Biomarkers: potential uses and limitations. NeuroRx.

[17] Committee on Biological Markers of the National Research Council (1987). Biological markers in environmental health research. Environ Health Perspect.

[18] Mastin JP, Henningsen GM, Fine LJ (1992). Use of biomarkers of occupational musculoskeletal disorders in epidemiology and laboratory animal model development. Scand J Work Environ Health.

[19] Saxton JM (2000). A review of current literature on physiological tests and soft tissue biomarkers applicable to work-related upper limb disorders. Occup Med (Lond).

[20] Gold JE, Hallman DM, Hellstrom F, Bjorklund M, Crenshaw AG, Djupsjobacka M et al. Systematic review of biochemical biomarkers for neck and upper-extremity musculoskeletal disorders. Scand J Work Environ Health. 2015. doi:10.5271/sjweh.3533. 10.5271/sjweh.3533 26599377

[21] Boocock MG, Collier JM, McNair PJ, Simmonds M, Larmer PJ, Armstrong B (2009). A framework for the classification and diagnosis of work-related upper extremity conditions: systematic review. Semin Arthritis Rheum.

[22] Organization WH (1985). Identification and control of work-related diseases.

[23] Seidenberg PH, Howe AH (2014). Musculoskeletal imaging: types and indications. Med Clin North Am.

[24] Aagesen AL, Melek M (2013). Choosing the right diagnostic imaging modality in musculoskeletal diagnosis. Prim Care.

[25] Bearcroft PW (2007). Imaging modalities in the evaluation of soft tissue complaints. Best Pract Res Clin Rheumatol.

[26] Bussieres AE, Peterson C, Taylor JA (2008). Diagnostic imaging guideline for musculoskeletal complaints in adults-an evidence-based approach-part 2: upper extremity disorders. J Manip Physiol Ther.

[27] Bruns DE, Huth EJ, Magid E, Young DS (2000). Toward a checklist for reporting of studies of diagnostic accuracy of medical tests. Clin Chem.

[28] Altman DG (1998). Statistical reviewing for medical journals. Stat Med.

[29] Downs SH, Black N (1998). The feasibility of creating a checklist for the assessment of the methodological quality both of randomised and non-randomised studies of health care interventions. J Epidemiol Community Health.

[30] Whiting P, Rutjes AW, Reitsma JB, Bossuyt PM, Kleijnen J (2003). The development of QUADAS: a tool for the quality assessment of studies of diagnostic accuracy included in systematic reviews. BMC Med Res Methodol.

[31] von Elm E, Altman DG, Egger M, Pocock SJ, Gotzsche PC, Vandenbroucke JP (2007). Strengthening the reporting of observational studies in epidemiology (STROBE) statement: guidelines for reporting observational studies. BMJ.

[32] Slavin RE (1995). Best evidence synthesis: an intelligent alternative to meta-analysis. J Clin Epidemiol.

[33] Mall NA, Kim HM, Keener JD, Steger-May K, Teefey SA, Middleton WD (2010). Symptomatic progression of asymptomatic rotator cuff tears: a prospective study of clinical and sonographic variables. J Bone Joint Surg Am.

[34] Keener JD, Galatz LM, Teefey SA, Middleton WD, Steger-May K, Stobbs-Cucchi G (2015). A prospective evaluation of survivorship of asymptomatic degenerative rotator cuff tears. J Bone Joint Surg Am.

[35] Keener JD, Hsu JE, Steger-May K, Teefey SA, Chamberlain AM, Yamaguchi K (2015). Patterns of tear progression for asymptomatic degenerative rotator cuff tears. J Shoulder Elb Surg.

[36] Moosmayer S, Tariq R, Stiris M, Smith HJ (2013). The natural history of asymptomatic rotator cuff tears: a three-year follow-up of fifty cases. J Bone Joint Surg Am.

[37] Dibai Filho AV, Packer AC, Costa AC, Berni-Schwarzenbeck KC, Rodrigues-Bigaton D (2012). Assessment of the upper trapezius muscle temperature in women with and without neck pain. J Manip Physiol Ther.

[38] Falla D, Jull G, Edwards S, Koh K, Rainoldi A (2004). Neuromuscular efficiency of the sternocleidomastoid and anterior scalene muscles in patients with chronic neck pain. Disabil Rehabil.

[39] Hirano Y, Sashi R, Izumi J, Itoi E, Watarai J (2006). Comparison of the MR findings on indirect MR arthrography in patients with rotator cuff tears with and without symptoms. Radiat Med.

[40] Kalra N, Seitz AL, Boardman ND, Michener LA (2010). Effect of posture on acromiohumeral distance with arm elevation in subjects with and without rotator cuff disease using ultrasonography. J Orthop Sports Phys Ther..

[41] O'Sullivan C, McCarthy Persson U, Blake C, Stokes M (2012). Rehabilitative ultrasound measurement of trapezius muscle contractile states in people with mild shoulder pain. Man Ther.

[42] Nilsen KB, Sand T, Westgaard RH, Stovner LJ, White LR, Bang Leistad R (2007). Autonomic activation and pain in response to low-grade mental stress in fibromyalgia and shoulder/neck pain patients. Eur J Pain.

[43] Strøm V, Røe C, Knardahl S (2009). Work-induced pain, trapezius blood flux, and muscle activity in workers with chronic shoulder and neck pain. Pain.

[44] Andersen LL, Blangsted AK, Nielsen PK, Hansen L, Vedsted P, Sjogaard G (2010). Effect of cycling on oxygenation of relaxed neck/shoulder muscles in women with and without chronic pain. Eur J Appl Physiol.

[45] Peolsson M, Larsson B, Brodin LA, Gerdle B (2008). A pilot study using tissue velocity ultrasound imaging (TVI) to assess muscle activity pattern in patients with chronic trapezius myalgia. BMC Musculoskelet Disord.

[46] Elliott J, Sterling M, Noteboom JT, Darnell R, Galloway G, Jull G (2008). Fatty infiltrate in the cervical extensor muscles is not a feature of chronic, insidious-onset neck pain. Clin Radiol.

[47] Fernandez-de-las-Penas C, Albert-Sanchis JC, Buil M, Benitez JC, Alburquerque-Sendin F (2008). Cross-sectional area of cervical multifidus muscle in females with chronic bilateral neck pain compared to controls. J Orthop Sports Phys Ther.

[48] Javanshir K, Rezasoltani A, Mohseni-Bandpei MA, Amiri M, Ortega-Santiago R, Fernandez-de-Las-Penas C (2011). Ultrasound assessment of bilateral longus colli muscles in subjects with chronic bilateral neck pain. Am J Phys Med Rehabil..

[49] Jesus-Moraleida FR, Ferreira PH, Pereira LS, Vasconcelos CM, Ferreira ML (2011). Ultrasonographic analysis of the neck flexor muscles in patients with chronic neck pain and changes after cervical spine mobilization. J Manip Physiol Ther.

[50] Park KN, Kwon OY, Choung SD, Kim SH (2013). Bilateral asymmetry of semispinalis capitis muscle thickness and neck motion during prone neck extension in subjects with unilateral posterior neck pain. J Phys Ther Sci.

[51] Sheard B, Elliott J, Cagnie B, O’Leary S (2012). Evaluating serratus anterior muscle function in neck pain using muscle functional magnetic resonance imaging. J Manip Physiol Ther.

[52] Rahnama L, Rezasoltani A, Zavieh MK, NooriKochi F, Baghban AA (2015). Differences in cervical multifidus muscle thickness during isometric contraction of shoulder muscles: a comparison between patients with chronic neck pain and healthy controls. J Manip Physiol Ther.

[53] Karimi N, Rezasoltani A, Rahnama L, Noori-Kochi F, Jaberzadeh S (2016). Ultrasonographic analysis of dorsal neck muscles thickness changes induced by isometric contraction of shoulder muscles: a comparison between patients with chronic neck pain and healthy controls. Man Ther.

[54] Biberthaler P, Wiedemann E, Nerlich A, Kettler M, Mussack T, Deckelmann S (2003). Microcirculation associated with degenerative rotator cuff lesions. In vivo assessment with orthogonal polarization spectral imaging during arthroscopy of the shoulder. J Bone Joint Surg Am.

[55] Funakoshi T, Iwasaki N, Kamishima T, Nishida M, Ito Y, Kondo M (2010). In vivo visualization of vascular patterns of rotator cuff tears using contrast-enhanced ultrasound. Am J Sports Med.

[56] Chang KV, Chen WS, Wang TG, Hung CY, Chien KL (2014). Quantitative ultrasound facilitates the exploration of morphological association of the long head biceps tendon with supraspinatus tendon full thickness tear. PLoS One.

[57] Choo HJ, Lee SJ, Kim DW, Park YM, Kim JH (2014). Assessment of the rotator cable in various rotator cuff conditions using indirect MR arthrography. Acta Radiol.

[58] Karthikeyan S, Griffin DR, Parsons N, Lawrence TM, Modi CS, Drew SJ (2015). Microvascular blood flow in normal and pathologic rotator cuffs. J Shoulder Elb Surg.

[59] Terabayashi N, Watanabe T, Matsumoto K, Takigami I, Ito Y, Fukuta M (2014). Increased blood flow in the anterior humeral circumflex artery correlates with night pain in patients with rotator cuff tear. J Orthop Sci.

[60] Cay N, Tosun O, Dogan M, Karaoglanoglu M, Bozkurt M (2012). The effect of morphometric relationship between the glenoid fossa and the humeral head on rotator cuff pathology. Acta Orthop Traumatol Turc.

[61] Rechardt M, Shiri R, Karppinen J, Jula A, Heliovaara M, Viikari-Juntura E (2010). Lifestyle and metabolic factors in relation to shoulder pain and rotator cuff tendinitis: a population-based study. BMC Musculoskelet Disord.

[62] Joensen J, Couppe C, Bjordal JM (2009). Increased palpation tenderness and muscle strength deficit in the prediction of tendon hypertrophy in symptomatic unilateral shoulder tendinopathy: an ultrasonographic study. Physiother.

[63] Arend CF, Arend AA, da Silva TR (2014). Diagnostic value of tendon thickness and structure in the sonographic diagnosis of supraspinatus tendinopathy: room for a two-step approach. Eur J Radiol.

[64] Li JQ, Tang KL, Wang J, Li QY, Xu HT, Yang HF (2011). MRI findings for frozen shoulder evaluation: is the thickness of the coracohumeral ligament a valuable diagnostic tool?. PLoS One.

[65] Song KD, Kwon JW, Yoon YC, Choi SH (2011). Indirect MR arthrographic findings of adhesive capsulitis. AJR Am J Roentgenol.

[66] Zhao W, Zheng X, Liu Y, Yang W, Amirbekian V, Diaz LE (2012). An MRI study of symptomatic adhesive capsulitis. PLoS One.

[67] Michelin P, Delarue Y, Duparc F, Dacher JN (2013). Thickening of the inferior glenohumeral capsule: an ultrasound sign for shoulder capsular contracture. Eur Radiol.

[68] Hebert LJ, Moffet H, Dufour M, Moisan C (2003). Acromiohumeral distance in a seated position in persons with impingement syndrome. J Magn Reson Imaging.

[69] Leong HT, Tsui S, Ying M, Leung VY, Fu SN (2012). Ultrasound measurements on acromio-humeral distance and supraspinatus tendon thickness: test-retest reliability and correlations with shoulder rotational strengths. J Sci Med Sport.

[70] Park JY, Hyun JK, Seo JB (2007). The effectiveness of digital infrared thermographic imaging in patients with shoulder impingement syndrome. J Shoulder Elb Surg.

[71] Daghir AA, Sookur PA, Shah S, Watson M (2012). Dynamic ultrasound of the subacromial-subdeltoid bursa in patients with shoulder impingement: a comparison with normal volunteers. Skelet Radiol.

[72] Tuite MJ, Petersen BD, Wise SM, Fine JP, Kaplan LD, Orwin JF (2007). Shoulder MR arthrography of the posterior labrocapsular complex in overhead throwers with pathologic internal impingement and internal rotation deficit. Skelet Radiol.

[73] Larsson R, Cai H, Zhang Q, Oberg PA, Larsson SE (1998). Visualization of chronic neck-shoulder pain: impaired microcirculation in the upper trapezius muscle in chronic cervico-brachial pain. Occup Med (Lond).

[74] Acero CO, Kuboki T, Maekawa K, Yamashita A, Clark GT (1999). Haemodynamic responses in chronically painful, human trapezius muscle to cold pressor stimulation. Arch Oral Biol.

[75] Cagnie B, Dhooge F, Van Akeleyen J, Cools A, Cambier D, Danneels L (2012). Changes in microcirculation of the trapezius muscle during a prolonged computer task. Eur J Appl Physiol.

[76] Flodgren GM, Crenshaw AG, Hellstrom F, Fahlstrom M (2010). Combining microdialysis and near-infrared spectroscopy for studying effects of low-load repetitive work on the intramuscular chemistry in trapezius myalgia. J Biomed Biotechnol.

[77] Sjogaard G, Rosendal L, Kristiansen J, Blangsted AK, Skotte J, Larsson B (2010). Muscle oxygenation and glycolysis in females with trapezius myalgia during stress and repetitive work using microdialysis and NIRS. Eur J Appl Physiol.

[78] Larsson B, Rosendal L, Kristiansen J, Sjogaard G, Sogaard K, Ghafouri B (2008). Responses of algesic and metabolic substances to 8 h of repetitive manual work in myalgic human trapezius muscle. Pain.

[79] Takiguchi S, Maekawa K, Ono T, Sasai N, Kaji M, Clark GT (2010). Relationship between a chronically painful trapezius muscle and its metabolic state analyzed with PET/CT. Oral Surg Oral Med Oral Pathol Oral Radiol Endod.

[80] Hallman DM, Lindberg LG, Arnetz BB, Lyskov E (2011). Effects of static contraction and cold stimulation on cardiovascular autonomic indices, trapezius blood flow and muscle activity in chronic neck-shoulder pain. Eur J Appl Physiol.

[81] Shiro Y, Arai YC, Matsubara T, Isogai S, Ushida T (2012). Effect of muscle load tasks with maximal isometric contractions on oxygenation of the trapezius muscle and sympathetic nervous activity in females with chronic neck and shoulder pain. BMC Musculoskelet Disord.

[82] Lund JP, Donga R, Widmer CG, Stohler CS (1991). The pain-adaptation model: a discussion of the relationship between chronic musculoskeletal pain and motor activity. Can J Physiol Pharmacol.

[83] O'Leary S, Jull G, Kim M, Vicenzino B (2007). Cranio-cervical flexor muscle impairment at maximal, moderate, and low loads is a feature of neck pain. Man Ther.

[84] Ylinen J, Salo P, Nykanen M, Kautiainen H, Hakkinen A (2004). Decreased isometric neck strength in women with chronic neck pain and the repeatability of neck strength measurements. Arch Phys Med Rehabil.

[85] Rosendal L, Larsson B, Kristiansen J, Peolsson M, Sogaard K, Kjaer M (2004). Increase in muscle nociceptive substances and anaerobic metabolism in patients with trapezius myalgia: microdialysis in rest and during exercise. Pain.

[86] Passatore M, Roatta S (2006). Influence of sympathetic nervous system on sensorimotor function: whiplash associated disorders (WAD) as a model. Eur J Appl Physiol.

[87] Maekawa K, Clark GT, Kuboki T (2002). Intramuscular hypoperfusion, adrenergic receptors, and chronic muscle pain. J Pain.

[88] Heiden M, Lyskov E, Djupsjobacka M, Hellstrom F, Crenshaw AG (2005). Effects of time pressure and precision demands during computer mouse work on muscle oxygenation and position sense. Eur J Appl Physiol.

[89] Elcadi GH, Forsman M, Crenshaw AG (2011). The relationship between oxygenation and myoelectric activity in the forearm and shoulder muscles of males and females. Eur J Appl Physiol.

[90] Sugisaki M, Misawa A, Ikai A, Young-Sung K, Tanabe H (2001). Sex differences in the hemoglobin oxygenation state of the resting healthy human masseter muscle. J Orofac Pain.

[91] Crenshaw AG, Elcadi GH, Hellstrom F, Mathiassen SE (2012). Reliability of near-infrared spectroscopy for measuring forearm and shoulder oxygenation in healthy males and females. Eur J Appl Physiol.

[92] Maikala RV, Bhambhani YN (2009). Microvascularity of the lumbar erector spinae muscle during sustained prone trunk extension test. Adv Exp Med Biol.

[93] Costes F, Denis C, Roche F, Prieur F, Enjolras F, Barthelemy JC (1999). Age-associated alteration of muscle oxygenation measured by near infrared spectroscopy during exercise. Arch Physiol Biochem.

[94] Neary JP (2004). Application of near infrared spectroscopy to exercise sports science. Can J Appl Physiol.

[95] Lindman R, Eriksson A, Thornell LE (1991). Fiber type composition of the human female trapezius muscle: enzyme-histochemical characteristics. Am J Anat.

[96] Zheng L, Siegmund G, Ozyigit G, Vasavada A (2013). Sex-specific prediction of neck muscle volumes. J Biomech.

[97] Rankin G, Stokes M, Newham DJ (2005). Size and shape of the posterior neck muscles measured by ultrasound imaging: normal values in males and females of different ages. Man Ther.

[98] Rudzki JR, Adler RS, Warren RF, Kadrmas WR, Verma N, Pearle AD (2008). Contrast-enhanced ultrasound characterization of the vascularity of the rotator cuff tendon: age- and activity-related changes in the intact asymptomatic rotator cuff. J Shoulder Elb Surg.

[99] Visser B, van Dieen JH (2006). Pathophysiology of upper extremity muscle disorders. J Electromyogr Kinesiol.

[100] Mulherin SA, Miller WC (2002). Spectrum bias or spectrum effect? Subgroup variation in diagnostic test evaluation. Ann Intern Med.

[101] Hegedus EJ, Cook C, Brennan M, Wyland D, Garrison JC, Driesner D (2010). Vascularity and tendon pathology in the rotator cuff: a review of literature and implications for rehabilitation and surgery. Br J Sports Med.

[102] Barbe MF, Gallagher S, Massicotte VS, Tytell M, Popoff SN, Barr-Gillespie AE (2013). The interaction of force and repetition on musculoskeletal and neural tissue responses and sensorimotor behavior in a rat model of work-related musculoskeletal disorders. BMC Musculoskelet Disord.

[103] Massicotte VS, Frara N, Harris MY, Amin M, Wade CK, Popoff SN (2015). Prolonged performance of a high repetition low force task induces bone adaptation in young adult rats, but loss in mature rats. Exp Gerontol.

[104] Barbe MF, Jain NX, Massicotte VS, Popoff SN, Barr-Gillespie AE (2015). Ergonomic task reduction prevents bone osteopenia in a rat model of upper extremity overuse. Ind Health.

[105] Jain NX, Barr-Gillespie AE, Clark BD, Kietrys DM, Wade CK, Litvin J (2014). Bone loss from high repetitive high force loading is prevented by ibuprofen treatment. J Musculoskelet Neuronal Interact.

[106] Saupe N, Pfirrmann CW, Schmid MR, Jost B, Werner CM, Zanetti M (2006). Association between rotator cuff abnormalities and reduced acromiohumeral distance. AJR Am J Roentgenol.

[107] Bigliani LU, Morrison DS, April EW (1986). The morphology of the acromion and its relationship to rotator cuff tears. Orthop Trans.

[108] Abramson RG, Burton KR, Yu JP, Scalzetti EM, Yankeelov TE, Rosenkrantz AB (2015). Methods and challenges in quantitative imaging biomarker development. Acad Radiol.

[109] Raunig DL, McShane LM, Pennello G, Gatsonis C, Carson PL, Voyvodic JT (2015). Quantitative imaging biomarkers: a review of statistical methods for technical performance assessment. Stat Methods Med Res.

[110] Sullivan DC, Obuchowski NA, Kessler LG, Raunig DL, Gatsonis C, Huang EP, et al. Metrology standards for quantitative imaging biomarkers. Radiol. 2015;142202 doi:10.1148/radiol.2015142202. 10.1148/radiol.2015142202 PMC4666097 26267831

[111] Richards RR, An KN, Bigliani LU, Friedman RJ, Gartsman GM, Gristina AG (1994). A standardized method for the assessment of shoulder function. J Shoulder Elb Surg.

[112] Vernon H, Mior S (1991). The neck disability index: a study of reliability and validity. J Manip Physiol Ther.

[113] Lippitt SB, Harryman DT, Matsen FA, Matsen FA, Fu FH, Hawkins RJ (1993). A practical tool for evaluation of function: the simple shoulder test. The shoulder: a balance of mobility and stability.

[114] Larsson R, Cai H, Zhang Q, Öberg P, Larsson S-E (1998). Visualization of chronic neck-shoulder pain: impaired microcirculation in the upper trapezius muscle in chronic cervico-brachial pain. Occup Med (Lond).

[115] Levy O, Relwani J, Zaman T, Even T, Venkateswaran B, Copeland S (2008). Measurement of blood flow in the rotator cuff using laser Doppler flowmetry. J Bone Joint Surg Br..

[116] Cay N, Tosun O, Isik C, Unal O, Kartal MG, Bozkurt M (2014). Is coracoacromial arch angle a predisposing factor for rotator cuff tears?. Diagn Interv Radiol.

[117] De Loose V, Van Den Oord M, Keser I, Burnotte F, Van Tiggelen D, Dumarey A (2009). MRI study of the morphometry of the cervical musculature in F-16 pilots. Aviat Space Environ Med.

[118] Gokalp G, Algin O, Yildirim N, Yazici Z (2011). Adhesive capsulitis: contrast-enhanced shoulder MRI findings. J Med Imaging Radiat Oncol.

[119] Kaymak B, Ozcakar L, Inanici F, Cetin A, Ariyurek M, Tan AA (2008). Forearm bone mineral density measurements in thoracic outlet syndrome. Rheumatol Int.

[120] Kim KC, Rhee KJ, Shin HD (2009). Adhesive capsulitis of the shoulder: dimensions of the rotator interval measured with magnetic resonance arthrography. J Shoulder Elb Surg.

[121] Lee MH, Ahn JM, Muhle C, Kim SH, Park JS, Kim SH (2003). Adhesive capsulitis of the shoulder: diagnosis using magnetic resonance arthrography, with arthroscopic findings as the standard. J Comput Assist Tomogr.

[122] Macarini L, Muscarella S, Lelario M, Stoppino L, Scalzo G, Scelzi A (2011). Rotator cable at MR imaging: considerations on morphological aspects and biomechanical role. Radiol Med.

[123] Manton GL, Schweitzer ME, Weishaupt D, Karasick D (2001). Utility of MR arthrography in the diagnosis of adhesive capsulitis. Skelet Radiol.

[124] Moses DA, Chang EY, Schweitzer ME (2006). The scapuloacromial angle: a 3D analysis of acromial slope and its relationship with shoulder impingement. J Magn Reson Imaging.

[125] O'Leary S, Cagnie B, Reeve A, Jull G, Elliott JM (2011). Is there altered activity of the extensor muscles in chronic mechanical neck pain? A functional magnetic resonance imaging study. Arch Phys Med Rehabil.

[126] Ahn KS, Kang CH, Oh YW, Jeong WK (2012). Correlation between magnetic resonance imaging and clinical impairment in patients with adhesive capsulitis. Skelet Radiol.

[127] Ballyns JJ, Turo D, Otto P, Shah JP, Hammond J, Gebreab T (2012). Office-based elastographic technique for quantifying mechanical properties of skeletal muscle. J Ultrasound Med.

[128] Brasseur JL, Lucidarme O, Tardieu M, Tordeur M, Montalvan B, Parier J (2004). Ultrasonographic rotator-cuff changes in veteran tennis players: the effect of hand dominance and comparison with clinical findings. Eur Radiol.

[129] Cholewinski JJ, Kusz DJ, Wojciechowski P, Cielinski LS, Zoladz MP (2008). Ultrasound measurement of rotator cuff thickness and acromio-humeral distance in the diagnosis of subacromial impingement syndrome of the shoulder. Knee Surg Sports Traumatol Arthrosc : official journal of the ESSKA.

[130] Curry EJ, Matzkin EE, Dong Y, Higgins LD, Katz JN, Jain NB (2015). Structural characteristics are not associated with pain and function in rotator cuff tears: the ROW cohort study. Orthop J Sports Med.

[131] Gokalp G, Yildirim N, Yazici Z, Ercan I (2010). Using chemical-shift MR imaging to quantify fatty degeneration within supraspinatus muscle due to supraspinatus tendon injuries. Skelet Radiol.

[132] Jeracitano D, Cooper RG, Lyon LJ, Jayson MI (1992). Abnormal temperature control suggesting sympathetic dysfunction in the shoulder skin of patients with frozen shoulder. Br J Rheumatol.

[133] Jung JY, Jee WH, Chun HJ, Kim YS, Chung YG, Kim JM (2006). Adhesive capsulitis of the shoulder: evaluation with MR arthrography. Eur Radiol.

[134] Lefevre-Colau MM, Drape JL, Fayad F, Rannou F, Diche T, Minvielle F (2005). Magnetic resonance imaging of shoulders with idiopathic adhesive capsulitis: reliability of measures. Eur Radiol.

[135] Roidis NT, Motamed S, Vaishnav S, Ebramzadeh E, Karachalios TS, Itamura JM (2009). The influence of the acromioclavicular joint degeneration on supraspinatus outlet impingement and the acromion shape. J Orthop Surg (Hong Kong).

[136] Tamai K, Yamato M (1997). Abnormal synovium in the frozen shoulder: a preliminary report with dynamic magnetic resonance imaging. J Shoulder Elb Surg.

[137] Toyoda H, Ito Y, Tomo H, Nakao Y, Koike T, Takaoka K (2005). Evaluation of rotator cuff tears with magnetic resonance arthrography. Clin Orthop Relat Res.

[138] Collinger JL, Fullerton B, Impink BG, Koontz AM, Boninger ML (2010). Validation of grayscale-based quantitative ultrasound in manual wheelchair users: relationship to established clinical measures of shoulder pathology. Am J Phys Med Rehabil.

[139] Dogan M, Cay N, Tosun O, Karaoglanoglu M, Bozkurt M (2012). Glenoid axis is not related with rotator cuff tears--a magnetic resonance imaging comparative study. Int Orthop.

[140] Girometti R, De Candia A, Sbuelz M, Toso F, Zuiani C, Bazzocchi M (2006). Supraspinatus tendon US morphology in basketball players: correlation with main pathologic models of secondary impingement syndrome in young overhead athletes. Preliminary report. Radiol Med.

[141] Huang CC, Ko SF, Ko JY, Huang HY, Ng SH, Wan YL (2005). Contracture of the deltoid muscle: sonographic evaluation with MRI correlation. AJR Am J Roentgenol.

[142] Kaneko K, DeMouy EH, Brunet ME (1994). MR evaluation of rotator cuff impingement: correlation with confirmed full-thickness rotator cuff tears. J Comput Assist Tomogr.

[143] Kim HM, Dahiya N, Teefey SA, Middleton WD, Stobbs G, Steger-May K (2010). Location and initiation of degenerative rotator cuff tears: an analysis of three hundred and sixty shoulders. J Bone Joint Surg Am.

[144] Rezasoltani A, Ali-Reza A, Khosro KK, Abbass R (2010). Preliminary study of neck muscle size and strength measurements in females with chronic non-specific neck pain and healthy control subjects. Man Ther.

[145] Rezasoltani A, Ahmadipoor A, Khademi-Kalantari K, Javanshir K (2012). The sign of unilateral neck semispinalis capitis muscle atrophy in patients with chronic non-specific neck pain. J Back Musculoskelet Rehabil.

[146] Shinozaki T, Takagishi K, Ichikawa A, Inoue T, Yamaji T, Ishikawa T (2003). Use of 2-[18F]-fluoro-2-deoxy-d-glucose positron emission tomography (FDG PET) imaging for the evaluation of muscle metabolic activity in ruptured rotator cuffs: identification of shoulder muscles by fusion imaging studies involving both FDG PET and magnetic resonance imaging. J Shoulder Elb Surg.

[147] Sundstrom T, Guez M, Hildingsson C, Toolanen G, Nyberg L, Riklund K (2006). Altered cerebral blood flow in chronic neck pain patients but not in whiplash patients: a 99mTc-HMPAO rCBF study. Eur Spine J.

[148] MacGillivray JD, Fealy S, Potter HG, O'Brien SJ (1998). Multiplanar analysis of acromion morphology. Am J Sports Med.

[149] Tracy MR, Trella TA, Nazarian LN, Tuohy CJ, Williams GR (2010). Sonography of the coracohumeral interval: a potential technique for diagnosing coracoid impingement. J Ultrasound Med.

[150] Vecchio PC, Adebajo AO, Chard MD, Thomas PP, Hazleman BL (1992). Thermography of frozen shoulder and rotator cuff tendinitis. Clin Rheumatol.

[151] Harrison MF, Neary JP, Albert WJ, Croll JC (2011). Neck pain and muscle function in a population of CH-146 helicopter aircrew. Aviat Space Environ Med.

[152] Ko JY, Huang CC, Chen WJ, Chen CE, Chen SH, Wang CJ (2006). Pathogenesis of partial tear of the rotator cuff: a clinical and pathologic study. J Shoulder Elb Surg.

[153] Larsson SE, Bodegard L, Henriksson KG, Oberg PA (1990). Chronic trapezius myalgia. Morphology and blood flow studied in 17 patients. Acta Orthop Scand.

[154] McGinley JC, Agrawal S, Biswal S (2012). Rotator cuff tears: association with acromion angulation on MRI. Clin Imaging.

[155] Wallny T, Wagner UA, Prange S, Schmitt O, Reich H (1999). Evaluation of chronic tears of the rotator cuff by ultrasound. A new index. J Bone Joint Surg Br.

[156] Leistad RB, Nilsen KB, Stovner LJ, Westgaard RH, Ro M, Sand T (2008). Similarities in stress physiology among patients with chronic pain and headache disorders: evidence for a common pathophysiological mechanism?. J Headache Pain.

[157] Di Mario M, Fraracci L (2005). MR study of the intrinsic acromial angle in 74 symptomatic patients. Radiol Med.

[158] Mani R, Cooper C, Kidd BL, Cole JD, Cawley MI (1989). Use of laser Doppler flowmetry and transcutaneous oxygen tension electrodes to assess local autonomic dysfunction in patients with frozen shoulder. J R Soc Med.

[159] Breidahl WH, Stafford Johnson DB, Newman JS, Adler RS (1998). Power Doppler sonography in tenosynovitis: significance of the peritendinous hypoechoic rim. J Ultrasound Med.

