# Supplementary material for: Systematic review of quantitative imaging biomarkers for neck and shoulder musculoskeletal disorders
Source: BMC Musculoskelet Disord. 2017 Sep 12;18:395. doi: 10.1186/s12891-017-1694-y (PMC5596923; doi:10.1186/s12891-017-1694-y)
Supplement: Supplementary file 1 — Search terms for musculoskeletal disorders (MSDs) and imaging markers. (PDF 14 kb) [file 12891_2017_1694_MOESM1_ESM.pdf]

**Additional file 1**. Search terms for musculoskeletal disorders (MSDs) and imaging markers.		
MSD		
	MESH terms	Upper extremity/pathology, upper extremity/physiopathology, rotator cuff, neck muscles, musculoskeletal pain, thoracic outlet syndrome, shoulder impingement syndrome, shoulder pain, cumulative trauma disorders, neck pain, musculoskeletal diseases tendinopathy, myofascial pain syndromes
	Keywords	Rotator cuff , musculoskeletal pain, thoracic outlet syndrome, shoulder impingement syndrome, frozen shoulder, adhesive capsulitis, shoulder pain, musculoskeletal disorder, MSD, muscle pain, neck pain, trapezius myalgia, neck-shoulder pain, musculoskeletal diseases, tendinopathy, tendonitis, tendinitis, tendinosis, tenosynovitis
Imaging marker		
	MESH terms	Biological markers, Diagnostic Imaging/methods, Laser-Doppler Flowmetry, Hemodynamics, skin temperature, Blood Circulation, Microcirculation, Regional Blood Flow, Galvanic Skin Response, Tendons/ultrasonography, Muscles/ultrasonography, Brachial

		Plexus/ultrasonography, Neck/ultrasonography, Upper Extremity/ultrasonography
	Keywords	Biological marker, biomarker, diffusion tensor imaging, near infrared spectroscopy, thermography, CT scan, PET scan, Positron emission tomography, functional magnetic resonance imaging, laser Doppler flowmetry, plethysmography, skin temperature, blood circulation, blood flow, microcirculation

Note: the search was restricted to include peer-reviewed articles between June 4, 1988 and October 14, 2016, English language, humans, and adults. Terms within each broad category (MSD, quantitative imaging marker) are combined with OR and between categories with AND.
