# Supplementary material for: Systematic review of quantitative imaging biomarkers for neck and shoulder musculoskeletal disorders
Source: BMC Musculoskelet Disord. 2017 Sep 12;18:395. doi: 10.1186/s12891-017-1694-y (PMC5596923; doi:10.1186/s12891-017-1694-y)
Supplement: Supplementary file 2 — Questions used in the primary screen for exclusion of articles. (PDF 9 kb) [file 12891_2017_1694_MOESM2_ESM.pdf]

**Additional file 2.** Questions used in the primary screen for exclusion of articles.
Exclusion questions
Case study?
Gray literature?
Review article?
Cadaver study?
Lower extremity MSD?
Widespread pain?
Traumatic onset?
Participants with known disc problems?
Conventional X-ray study?
Experiment which induce pain in healthy subjects or those with MSDs by injecting substances into the body?

Notes: An answer "yes" to one or more questions led to exclusion. Two independent reviewers had to agree on these items in order to exclude a study.
