# Supplementary material for: Systematic review of quantitative imaging biomarkers for neck and shoulder musculoskeletal disorders
Source: BMC Musculoskelet Disord. 2017 Sep 12;18:395. doi: 10.1186/s12891-017-1694-y (PMC5596923; doi:10.1186/s12891-017-1694-y)
Supplement: Supplementary file 3 — Questions used in the secondary screen for quality assessment. (PDF 13 kb) [file 12891_2017_1694_MOESM3_ESM.pdf]

**Additional file 3**. Questions used in the secondary screen for quality assessment.	
1.	Was the hypothesis/aim/objective clearly stated?
2.	Was the biomarker described in sufficient detail to permit its replication?
3.	Was the MSD definition or diagnostic criteria clearly described?
4.	Was the MSD symptom severity clearly described?
5.	Was there a comparison group?
6.	Were the selection criteria (inclusion/exclusion) clearly described?
7.	Was the participation/response rate to the initial invitation 65% or greater?
8.	Was loss to follow up less than 35%?
9.	Was an attempt made to blind those analyzing the biomarker to case condition?
10.	Was the time period between assessment of case condition and biomarker assessment short enough to be reasonably sure that the case condition did not change?
11.	Was there any control for confounding either through restriction of subjects or through adjustment in the analysis?
12.	Was the distribution of principal confounders in each group of subjects clearly described?
13.	Was there a power analysis to find an adequate sample size?
14.	Were the statistical models used to assess the main outcomes adequately described?
15.	Was the statistical analysis appropriate?
16.	Were estimates of the random variability in the main effect reported?
17.	Were the main findings clearly described?

Note: Possible responses were "yes", "unknown/not applicable" or "no".
