# Supplementary material for: Systematic review of quantitative imaging biomarkers for neck and shoulder musculoskeletal disorders
Source: BMC Musculoskelet Disord. 2017 Sep 12;18:395. doi: 10.1186/s12891-017-1694-y (PMC5596923; doi:10.1186/s12891-017-1694-y)
Supplement: Supplementary file 4 — PubMed search string. (PDF 5 kb) [file 12891_2017_1694_MOESM4_ESM.pdf]

Additional File 4. PubMed search string.

("Upper Extremity/pathology"[Mesh] OR "Upper Extremity/physiopathology"[Mesh] OR "Rotator Cuff"[Mesh] OR "rotator cuff"[Title/Abstract] OR "Neck Muscles"[Mesh] OR "Musculoskeletal Pain"[Mesh:noexp] OR "musculoskeletal pain"[Title/Abstract] OR "Thoracic Outlet Syndrome"[Mesh:noexp] OR "thoracic outlet syndrome"[Title/Abstract] OR "Shoulder Impingement Syndrome"[Mesh] OR "shoulder impingement syndrome"[Title/Abstract] OR "frozen shoulder"[Title/Abstract] OR "adhesive capsulitis"[Title/Abstract] OR "Shoulder Pain"[Mesh] OR "shoulder pain"[Title/Abstract] OR "Cumulative trauma disorders"[Mesh] OR "musculoskeletal disorder"[Title/Abstract] OR "MSD"[Title/Abstract] OR "muscle pain"[Title/Abstract] OR "Neck pain"[Mesh] OR "neck pain"[Title/Abstract] OR "trapezius myalgia"[Title/Abstract] OR "neck-shoulder pain"[Title/Abstract] OR "Musculoskeletal Diseases"[Mesh:noexp] OR "musculoskeletal diseases"[Title/Abstract] OR "Tendinopathy"[Mesh] OR "tendinopathy"[Title/Abstract] OR "tendonitis"[Title/Abstract] OR "tendinitis"[Title/Abstract] OR "tendinosis"[Title/Abstract] OR "tenosynovitis"[Title/Abstract] OR "Myofascial Pain Syndromes"[Mesh]) AND ("Biological Markers"[Mesh:noexp] OR "Biological marker"[Title/Abstract] OR "Biomarker"[Title/Abstract] OR "Diagnostic Imaging/methods"[Mesh] OR "Diffusion tensor imaging"[Title/Abstract] OR "Near infrared spectroscopy"[Title/Abstract] OR "Thermography"[Title/Abstract] OR "CT scan"[Title/Abstract] OR "PET scan"[Title/Abstract] OR "Positron emission tomography"[Title/Abstract] OR "Functional magnetic resonance imaging"[Title/Abstract] OR "Laser-Doppler Flowmetry"[Mesh] OR "Laser doppler flowmetry"[Title/Abstract] OR "plethysmography"[Title/Abstract] OR "Hemodynamics"[Mesh] OR "skin temperature"[Mesh] OR "Skin temperature"[Title/Abstract] OR "Blood Circulation"[Mesh:noexp] OR "Blood circulation"[Title/Abstract] OR "Blood flow"[Title/Abstract] OR "Microcirculation"[Title/Abstract] OR "Microcirculation"[Mesh] OR "Regional Blood Flow"[Mesh] OR "Galvanic Skin Response"[Mesh] OR "Tendons/ultrasonography"[Mesh] OR "Muscles/ultrasonography"[Mesh] OR "Brachial Plexus/ultrasonography"[Mesh] OR "Neck/ultrasonography"[Mesh] OR "Upper Extremity/ultrasonography"[Mesh]) AND (("1988/06/04"[PDAT] : "2016/10/14"[PDAT]) AND "humans"[MeSH Terms] AND English[lang] AND "adult"[MeSH Terms])
