# Supplementary material for: Systematic review of quantitative imaging biomarkers for neck and shoulder musculoskeletal disorders
Source: BMC Musculoskelet Disord. 2017 Sep 12;18:395. doi: 10.1186/s12891-017-1694-y (PMC5596923; doi:10.1186/s12891-017-1694-y)
Supplement: Supplementary file 5 — Data extraction items. (PDF 9 kb) [file 12891_2017_1694_MOESM5_ESM.pdf]

Additional File 5. Data extraction items.

1. What is the hypothesis/aim/objective of the study?

2. What is the biomarker(s) of interest?

    2a. What is the location of the body tissue from which it was imaged?

3. Describe any experimental challenge during which biomarkers and/or MSD severity was assessed.

4. What is the MSD/pain outcome(s) of interest?

    4a. How is symptom/case severity described? State the average severity and severity range, if given.

    4b. What is the duration of symptoms/MSD? State the average duration and duration range, if given.

5. Is there a control group in the study?

    5a. Is the study longitudinal?

6. What is the study population from which potential participants were selected from? Specify for cases and controls.

    6a. What inclusion and/or exclusion criteria were used?

    6b. What was the size of the study population – specify for cases and comparison group (if there is one)?

    6c. What is the mean age and range (or SD) of cases and controls?

    6d. Which genders are study participants?

    6e. What was the participation/response rate?

7. State results in terms of effect sizes and precision (ex., OR = 3.2 [95% CI: 2.2-4.6]), if possible.

    7a. Is there at least one statistically significant association between MSD(s) and biomarker(s) in the study?
