# Supplementary material for: Systematic review of quantitative imaging biomarkers for neck and shoulder musculoskeletal disorders
Source: BMC Musculoskelet Disord. 2017 Sep 12;18:395. doi: 10.1186/s12891-017-1694-y (PMC5596923; doi:10.1186/s12891-017-1694-y)
Supplement: Supplementary file 6 — Quality scores for each of the reviewed papers in the primary screen, including the papers of sufficient quality (≥70%) and insufficient quality (< 70%) [114–159]. (DOCX 177 kb) [file 12891_2017_1694_MOESM6_ESM.docx]

| **Additional file 6**. Quality scores for each of the reviewed papers in the primary screen, including the papers of sufficient quality (≥ 70%) and insufficient quality (< 70%). | | | | | | | | | |
| --- | --- | --- | --- | --- | --- | --- | --- | --- | --- |
|  | | | | | | | | | |
| Criteria (see additional file 3), Primary author | Aim | Biomarker  described | MSD  definition  described | MSD  severity  described | Comparison  group | Selection  criteria  described | Response  Rate > 65% | Loss to  follow  up < 35% | Blinded  analyses |
| Sufficient quality (above median) |  |  |  |  |  |  |  |  |  |
| Choo (2014) [57] | 1 | 1 | 1 | 1 | 1 | 1 | 1 | 0 | 1 |
| Keener (2015) [34] | 1 | 1 | 1 | 1 | 1 | 1 | 0 | 1 | 0 |
| Li (2011) [64] | 1 | 1 | 1 | 1 | 1 | 1 | 1 | 0 | 1 |
| Cay (2012) [60] | 1 | 1 | 1 | 0 | 1 | 1 | 1 | 0 | 1 |
| Javanshir (2011) [48] | 1 | 1 | 1 | 1 | 1 | 1 | 0 | 0 | 1 |
| Moosmayer (2013) [36] | 1 | 1 | 1 | 1 | 1 | 1 | 0 | 1 | 1 |
| Sjøgaard (2010) [77] | 1 | 1 | 1 | 1 | 1 | 1 | 1 | 0 | 0 |
| Strøm (2009) [43] | 1 | 1 | 1 | 1 | 1 | 1 | 0 | 1 | 0 |
| Tuite (2007) [72] | 1 | 1 | 1 | 1 | 1 | 1 | 1 | 0 | 1 |
| Arend (2013) [63] | 1 | 1 | 1 | 0 | 1 | 1 | 1 | 0 | 1 |
| Fernández-de-las-Peñas (2008) [47] | 1 | 1 | 1 | 1 | 1 | 0 | 0 | 0 | 1 |
| Funakoshi (2010) [55] | 1 | 1 | 1 | 1 | 1 | 1 | 0 | 0 | 1 |
| Hallman (2011) [80] | 1 | 1 | 1 | 1 | 1 | 1 | 0 | 0 | 0 |
| Jesus-Moraleida (2011) [49] | 1 | 1 | 1 | 1 | 1 | 1 | 0 | 0 | 0 |
| Kalra (2010) [40] | 1 | 1 | 1 | 1 | 1 | 1 | 0 | 0 | 1 |
| Keener (2015) [35] | 1 | 1 | 1 | 0 | 1 | 1 | 0 | 1 | 0 |
| Leong (2012) [69] | 1 | 1 | 1 | 1 | 1 | 0 | 0 | 0 | 1 |
| Mall (2010) [33] | 1 | 1 | 1 | 1 | 1 | 1 | 1 | 0 | 0 |
| Nilsen (2007) [42] | 1 | 1 | 1 | 1 | 1 | 1 | 0 | 0 | 1 |
| O'Sullivan (2012) [41] | 1 | 1 | 1 | 1 | 1 | 1 | 0 | 0 | 1 |
| Park (2007) [70] | 1 | 1 | 1 | 1 | 1 | 1 | 0 | 0 | 0 |
| Peolsson (2008) [45] | 1 | 1 | 1 | 1 | 1 | 1 | 0 | 0 | 0 |
| Song (2011) [65] | 1 | 1 | 1 | 1 | 1 | 1 | 0 | 0 | 1 |
| Takiguchi (2010) [79] | 1 | 1 | 1 | 1 | 1 | 1 | 1 | 0 | 1 |
| Terabayashi (2014) [59] | 1 | 1 | 1 | 0 | 1 | 1 | 1 | 0 | 0 |
| Acero (1999) [74] | 1 | 1 | 1 | 1 | 1 | 1 | 0 | 0 | 0 |
| Andersen (2010) [44] | 1 | 1 | 1 | 1 | 1 | 1 | 0 | 0 | 0 |
| Biberthaler (2003) [54] | 1 | 1 | 1 | 0 | 0 | 1 | 0 | 0 | 1 |
| Cagnie (2012) [75] | 1 | 1 | 1 | 1 | 1 | 1 | 0 | 0 | 0 |
| Chang (2014) [56] | 1 | 1 | 1 | 0 | 1 | 1 | 0 | 0 | 0 |
| Daghir (2011) [71] | 1 | 1 | 1 | 1 | 1 | 1 | 0 | 0 | 0 |
| Dibai Filho (2012) [37] | 1 | 1 | 0 | 0 | 1 | 1 | 0 | 0 | 1 |
| Elliott (2008) [46] | 1 | 1 | 1 | 1 | 1 | 1 | 0 | 0 | 0 |
| Falla (2004) [38] | 1 | 1 | 1 | 1 | 1 | 1 | 0 | 0 | 0 |
| Flodgren (2010) [76] | 1 | 1 | 1 | 1 | 0 | 1 | 0 | 0 | 1 |
| Hébert (2003) [68] | 1 | 1 | 1 | 1 | 1 | 1 | 0 | 0 | 1 |
| Hirano (2006) [39] | 1 | 1 | 1 | 1 | 1 | 1 | 1 | 0 | 0 |
| Joensen (2009) [62] | 1 | 1 | 1 | 1 | 1 | 1 | 0 | 0 | 0 |
| Karimi (2016) [53] | 1 | 1 | 1 | 0 | 1 | 1 | 1 | 0 | 0 |
| Karthikeyan (2015) [58] | 1 | 1 | 1 | 0 | 1 | 1 | 0 | 0 | 0 |
| Larsson (1998) [114] | 1 | 1 | 1 | 1 | 1 | 0 | 1 | 0 | 0 |
| Levy (2008) [115] | **1** | 1 | **1** | 1 | 1 | **0** | 1 | **0** | 0 |
| Michelin (2013) [67] | 1 | 1 | 1 | 0 | 1 | 1 | 1 | 0 | 0 |
| Park (2013) [50] | 1 | 1 | 1 | 1 | 1 | 1 | 0 | 0 | 0 |
| Rahnama (2015) [52] | 1 | 1 | 1 | 1 | 1 | 1 | 0 | 0 | 0 |
| Rechardt (2010) [61] | 1 | 1 | 1 | 1 | 1 | 1 | 1 | 0 | 0 |
| Sheard (2012) [51] | 1 | 1 | 1 | 1 | 1 | 1 | 0 | 0 | 0 |
| Shiro (2012) [81] | 1 | 1 | 1 | 1 | 1 | 1 | 0 | 0 | 0 |
| Zhao (2012) [66] | 1 | 1 | 1 | 1 | 1 | 1 | 0 | 0 | 1 |
|  | | | | | | | | | |
| Insufficient quality (below median) |  |  |  |  |  |  |  |  |  |
| Cay (2014) [116] | 1 | 1 | 1 | 0 | 1 | 1 | 0 | 0 | 0 |
| De Loose (2009) [117] | 1 | 1 | 1 | 1 | 1 | 1 | 0 | 0 | 1 |
| Gokalp (2011) [118] | 1 | 1 | 1 | 1 | 1 | 1 | 0 | 0 | 1 |
| Kaymak (2008) [119] | 1 | 0 | 0 | 0 | 1 | 1 | 1 | 0 | 0 |
| Kim (2009) [120] | 1 | 1 | 1 | 1 | 1 | 1 | 0 | 0 | 0 |
| Lee (2003) [121] | 1 | 1 | 1 | 0 | 1 | 0 | 0 | 0 | 1 |
| Macarini (2010) [122] | 1 | 1 | 0 | 0 | 1 | 1 | 0 | 0 | 1 |
| Manton (2001) [123] | 1 | 1 | 1 | 0 | 1 | 0 | 0 | 0 | 1 |
| Moses (2006) [124] | 1 | 1 | 1 | 0 | 1 | 1 | 0 | 0 | 0 |
| O'Leary (2011) [125] | 1 | 1 | 1 | 1 | 1 | 1 | 0 | 0 | 0 |
| Ahn (2012) [126] | 1 | 1 | 0 | 0 | 0 | 1 | 1 | 0 | 0 |
| Ballyns (2012) [127] | 1 | 1 | 1 | 1 | 1 | 1 | 0 | 0 | 1 |
| Brasseur (2003) [128] | 1 | 1 | 1 | 1 | 1 | 0 | 0 | 0 | 0 |
| Cholewinski (2007) [129] | 1 | 1 | 1 | 1 | 1 | 1 | 0 | 0 | 0 |
| Curry (2013) [130] | 1 | 1 | 1 | 1 | 1 | 1 | 0 | 0 | 1 |
| Gokalp (2010) [131] | 1 | 1 | 1 | 1 | 1 | 0 | 0 | 0 | 0 |
| Jeracitano (1992) [132] | 0 | 1 | 1 | 1 | 1 | 1 | 0 | 0 | 0 |
| Jung (2005) [133] | 1 | 1 | 0 | 0 | 1 | 1 | 0 | 0 | 1 |
| Lefevre-Colau (2005) [134] | 1 | 1 | 1 | 1 | 1 | 1 | 0 | 0 | 1 |
| Roidis (2009) [135] | 1 | 1 | 1 | 1 | 1 | 0 | 0 | 0 | 0 |
| Tamai (1997) [136] | 0 | 1 | 1 | 1 | 1 | 1 | 0 | 0 | 0 |
| Toyoda (2005) [137] | 1 | 1 | 1 | 1 | 0 | 0 | 0 | 0 | 1 |
| Collinger (2010) [138] | 1 | 1 | 1 | 1 | 1 | 1 | 0 | 0 | 0 |
| Dogan (2011) [139] | 1 | 1 | 0 | 0 | 1 | 0 | 0 | 0 | 0 |
| Girometti (2006) [140] | 1 | 1 | 0 | 0 | 1 | 0 | 0 | 0 | 0 |
| Huang (2005) [141] | 1 | 1 | 1 | 1 | 0 | 1 | 0 | 0 | 0 |
| Kaneko (1994) [142] | 1 | 1 | 1 | 1 | 1 | 1 | 0 | 0 | 1 |
| Kim (2010) [143] | 1 | 1 | 1 | 0 | 0 | 1 | 0 | 0 | 0 |
| Rezasoltani (2010) [144] | 1 | 1 | 0 | 0 | 1 | 0 | 0 | 0 | 0 |
| Rezasoltani (2012) [145] | 1 | 1 | 1 | 1 | 1 | 1 | 0 | 0 | 0 |
| Shinozaki (2003) [146] | 0 | 1 | 1 | 1 | 1 | 1 | 0 | 0 | 0 |
| Sundström (2006) [147] | 1 | 1 | 1 | 1 | 1 | 0 | 0 | 0 | 0 |
| MacGillivray (1998) [148] | 1 | 1 | 1 | 1 | 1 | 0 | 1 | 0 | 0 |
| Tracy (2010) [149] | 1 | 1 | 1 | 1 | 1 | 0 | 0 | 0 | 0 |
| Vecchio (1992) [150] | 1 | 1 | 1 | 1 | 1 | 1 | 0 | 0 | 1 |
| Harrison (2011) [151] | 1 | 1 | 0 | 0 | 1 | 0 | 0 | 0 | 0 |
| Ko (2006) [152] | 1 | 0 | 1 | 1 | 1 | 0 | 0 | 0 | 1 |
| Larsson (1990) [153] | 1 | 1 | 1 | 1 | 0 | 0 | 0 | 0 | 0 |
| McGinley (2012) [154] | 1 | 1 | 0 | 0 | 1 | 0 | 0 | 0 | 1 |
| Wallny (1999) [155] | 0 | 1 | 1 | 1 | 1 | 1 | 0 | 0 | 0 |
| Leistad (2008) [156] | 1 | 0 | 0 | 0 | 1 | 0 | 0 | 0 | 0 |
| Di Mario (2005) [157] | 1 | 1 | 0 | 0 | 1 | 0 | 0 | 0 | 0 |
| Mani (1989) [158] | 0 | 0 | 1 | 1 | 1 | 1 | 0 | 0 | 0 |
| Breidahl (1998) [159] | 1 | 1 | 0 | 0 | 1 | 0 | 0 | 0 | 0 |
|  |  |  |  |  |  |  |  |  |  |
| Percentage of ”yes” scores | 95 | 96 | 86 | 71 | 92 | 75 | 19 | 4 | 37 |

| **Additional file 6**. (cont.) | | | | | | | | | |
| --- | --- | --- | --- | --- | --- | --- | --- | --- | --- |
| Criteria (see additional file 3), Primary author | Time- period  short enough | Control  for confound  ing | Principal confound  ers  described | Power analyses | Statistical methods  described | Statistical  Analyses  Appropriate | Estimates of variability | Findings clearly described | **Total score** |
| Sufficient quality (above median) |  |  |  |  |  |  |  |  |  |
| Choo (2014) [[57](#_ENREF_57)] | 1 | 1 | 1 | 0 | 1 | 1 | 1 | 1 | 15 |
| Keener (2015) [[34](#_ENREF_34)] | 1 | 1 | 1 | 1 | 1 | 1 | 1 | 1 | 15 |
| Li (2011) [[64](#_ENREF_64)] | 1 | 1 | 1 | 0 | 1 | 1 | 1 | 1 | 15 |
| Cay (2012) [[60](#_ENREF_60)] | 1 | 1 | 1 | 0 | 1 | 1 | 1 | 1 | 14 |
| Javanshir (2011) [[48](#_ENREF_48)] | 0 | 1 | 1 | 1 | 1 | 1 | 1 | 1 | 14 |
| Moosmayer (2013) [[36](#_ENREF_36)] | 0 | 0 | 1 | 1 | 1 | 1 | 1 | 1 | 14 |
| Sjøgaard (2009) [[77](#_ENREF_76)] | 1 | 1 | 1 | 0 | 1 | 1 | 1 | 1 | 14 |
| Strøm (2009) [[43](#_ENREF_43)] | 1 | 1 | 1 | 0 | 1 | 1 | 1 | 1 | 14 |
| Tuite (2007) [[72](#_ENREF_71)] | 1 | 0 | 1 | 0 | 1 | 1 | 1 | 1 | 14 |
| Arend (2014) [[63](#_ENREF_63)] | 1 | 0 | 1 | 0 | 1 | 1 | 1 | 1 | 13 |
| Fernández-de-las-Peñas (2008) [[47](#_ENREF_47)] | 0 | 1 | 1 | 1 | 1 | 1 | 1 | 1 | 13 |
| Funakoshi (2010) [[55](#_ENREF_55)] | 1 | 0 | 1 | 0 | 1 | 1 | 1 | 1 | 13 |
| Hallman (2011) [80] | 1 | 1 | 1 | 0 | 1 | 1 | 1 | 1 | 13 |
| Jesus-Moraleida (2011) [[49](#_ENREF_49)] | 0 | 1 | 1 | 1 | 1 | 1 | 1 | 1 | 13 |
| Kalra (2010) [[40](#_ENREF_40)] | 0 | 0 | 1 | 1 | 1 | 1 | 1 | 1 | 13 |
| Keener (2015) [[35](#_ENREF_35)] | 1 | 1 | 0 | 1 | 1 | 1 | 1 | 1 | 13 |
| Leong (2011) [[69](#_ENREF_69)] | 1 | 1 | 1 | 0 | 1 | 1 | 1 | 1 | 13 |
| Mall (2010) [[33](#_ENREF_33)] | 1 | 0 | 1 | 0 | 1 | 1 | 1 | 1 | 13 |
| Nilsen (2007) [[42](#_ENREF_42)] | 0 | 1 | 1 | 0 | 1 | 1 | 1 | 1 | 13 |
| O'Sullivan (2012) [[41](#_ENREF_41)] | 1 | 0 | 1 | 0 | 1 | 1 | 1 | 1 | 13 |
| Park (2007) [[70](#_ENREF_70)] | 1 | 1 | 1 | 0 | 1 | 1 | 1 | 1 | 13 |
| Peolsson (2008) [[45](#_ENREF_45)] | 1 | 1 | 1 | 0 | 1 | 1 | 1 | 1 | 13 |
| Song (2011) [[65](#_ENREF_65)] | 0 | 1 | 1 | 0 | 1 | 1 | 1 | 1 | 13 |
| Takiguchi (2010) [[79](#_ENREF_78)] | 0 | 1 | 0 | 0 | 1 | 1 | 1 | 1 | 13 |
| Terabayashi (2014) [[59](#_ENREF_59)] | 1 | 1 | 1 | 0 | 1 | 1 | 1 | 1 | 13 |
| Acero (1999) [[74](#_ENREF_73)] | 0 | 1 | 1 | 0 | 1 | 1 | 1 | 1 | 12 |
| Andersen (2010) [[44](#_ENREF_44)] | 0 | 1 | 1 | 0 | 1 | 1 | 1 | 1 | 12 |
| Biberthaler (2003) [[54](#_ENREF_54)] | 1 | 1 | 1 | 0 | 1 | 1 | 1 | 1 | 12 |
| Cagnie (2012) [[75](#_ENREF_74)] | 1 | 0 | 1 | 0 | 1 | 1 | 1 | 1 | 12 |
| Chang (2014) [[56](#_ENREF_56)] | 1 | 1 | 1 | 0 | 1 | 1 | 1 | 1 | 12 |
| Daghir (2011) [71] | 0 | 1 | 1 | 1 | 1 | 0 | 1 | 1 | 12 |
| Dibai Filho (2012) [[37](#_ENREF_37)] | 0 | 1 | 1 | 1 | 1 | 1 | 1 | 1 | 12 |
| Elliott (2008) [[46](#_ENREF_46)] | 0 | 1 | 1 | 0 | 1 | 1 | 1 | 1 | 12 |
| Falla (2004) [[38](#_ENREF_38)] | 0 | 1 | 1 | 0 | 1 | 1 | 1 | 1 | 12 |
| Flodgren (2010) [[76](#_ENREF_75)] | 1 | 1 | 0 | 0 | 1 | 1 | 1 | 1 | 12 |
| Hébert (2003) [[68](#_ENREF_68)] | 0 | 0 | 1 | 0 | 1 | 1 | 1 | 1 | 12 |
| Hirano (2006) [[39](#_ENREF_39)] | 1 | 0 | 1 | 0 | 1 | 1 | 0 | 1 | 12 |
| Joensen (2009) [[62](#_ENREF_62)] | 1 | 0 | 1 | 0 | 1 | 1 | 1 | 1 | 12 |
| Karimi (2016) [[53](#_ENREF_53)] | 0 | 1 | 1 | 0 | 1 | 1 | 1 | 1 | 12 |
| Karthikeyan (2015) [[58](#_ENREF_58)] | 1 | 1 | 1 | 0 | 1 | 1 | 1 | 1 | 12 |
| Larsson (1998) [11[4](#_ENREF_116)] | 1 | 1 | 0 | 0 | 1 | 1 | 1 | 1 | 12 |
| Levy (2008) [[11](#_ENREF_117)5] | 1 | 1 | 1 | 0 | 1 | 0 | 1 | 1 | 12 |
| Michelin (2013) [[67](#_ENREF_67)] | 1 | 0 | 1 | 0 | 1 | 1 | 1 | 1 | 12 |
| Park (2013) [[50](#_ENREF_50)] | 0 | 1 | 1 | 0 | 1 | 1 | 1 | 1 | 12 |
| Rahnama (2015) [[52](#_ENREF_52)] | 0 | 1 | 1 | 0 | 1 | 1 | 1 | 1 | 12 |
| Rechardt (2010) [[61](#_ENREF_61)] | 0 | 1 | 1 | 0 | 1 | 1 | 0 | 1 | 12 |
| Sheard (2012) [[51](#_ENREF_51)] | 0 | 1 | 1 | 0 | 1 | 1 | 1 | 1 | 12 |
| Shiro (2012) [[8](#_ENREF_80)1] | 0 | 1 | 1 | 0 | 1 | 1 | 1 | 1 | 12 |
| Zhao (2012) [[66](#_ENREF_66)] | 0 | 1 | 1 | 0 | 1 | 1 | 0 | 1 | 12 |
|  |  |  |  |  |  |  |  |  |  |
| Insufficient quality (below median) |  |  |  |  |  |  |  |  |  |
| Cay (2014) [[11](#_ENREF_118)6] | 1 | 0 | 1 | 0 | 1 | 1 | 1 | 1 | 11 |
| De Loose (2009) [[11](#_ENREF_119)7] | 0 | 1 | 0 | 0 | 1 | 1 | 1 | 1 | 11 |
| Gokalp (2011) [[1](#_ENREF_120)18] | 0 | 0 | 1 | 0 | 1 | 0 | 1 | 1 | 11 |
| Kaymak (2008) [[1](#_ENREF_121)19] | 1 | 1 | 1 | 0 | 1 | 1 | 1 | 1 | 11 |
| Kim (2009) [[12](#_ENREF_122)0] | 1 | 0 | 1 | 0 | 1 | 1 | 0 | 1 | 11 |
| Lee (2003) [[12](#_ENREF_123)1] | 0 | 1 | 1 | 0 | 1 | 1 | 1 | 1 | 11 |
| Macarini (2010) [[12](#_ENREF_124)2] | 0 | 1 | 1 | 0 | 1 | 1 | 1 | 1 | 11 |
| Manton (2001) [[12](#_ENREF_125)3] | 1 | 0 | 1 | 0 | 1 | 1 | 1 | 1 | 11 |
| Moses (2006) [[12](#_ENREF_126)4] | 1 | 0 | 1 | 0 | 1 | 1 | 1 | 1 | 11 |
| O'Leary (2011) [[12](#_ENREF_127)5] | 0 | 0 | 1 | 0 | 1 | 1 | 1 | 1 | 11 |
| Ahn (2012) [[12](#_ENREF_128)6] | 1 | 1 | 0 | 0 | 1 | 1 | 1 | 1 | 10 |
| Ballyns (2012) [[12](#_ENREF_129)7] | 1 | 0 | 1 | 0 | 1 | 0 | 0 | 0 | 10 |
| Brasseur (2003) [[1](#_ENREF_130)28] | 0 | 1 | 1 | 0 | 1 | 0 | 1 | 1 | 10 |
| Cholewinski (2007) [[1](#_ENREF_131)29] | 0 | 0 | 1 | 0 | 1 | 1 | 1 | 0 | 10 |
| Curry (2013) [[13](#_ENREF_132)0] | 0 | 1 | 0 | 0 | 0 | 0 | 1 | 1 | 10 |
| Gokalp (2010) [[13](#_ENREF_133)1] | 1 | 0 | 0 | 0 | 1 | 1 | 1 | 1 | 10 |
| Jeracitano (1992) [[13](#_ENREF_134)2] | 1 | 1 | 1 | 0 | 1 | 0 | 1 | 0 | 10 |
| Jung (2005) [[13](#_ENREF_135)3] | 0 | 0 | 1 | 0 | 1 | 1 | 1 | 1 | 10 |
| Lefevre-Colau (2005) [[13](#_ENREF_136)4] | 0 | 0 | 0 | 0 | 1 | 0 | 1 | 1 | 10 |
| Roidis (2009) [[13](#_ENREF_137)5] | 1 | 0 | 1 | 0 | 1 | 1 | 0 | 1 | 10 |
| Tamai (1997) [[13](#_ENREF_138)6] | 0 | 0 | 1 | 0 | 1 | 1 | 1 | 1 | 10 |
| Toyoda (2005) [[13](#_ENREF_139)7] | 1 | 1 | 1 | 0 | 0 | 0 | 1 | 1 | 10 |
| Collinger (2010) [[1](#_ENREF_140)38] | 0 | 0 | 0 | 0 | 1 | 0 | 1 | 1 | 9 |
| Dogan (2011) [[1](#_ENREF_141)39] | 1 | 0 | 1 | 0 | 1 | 1 | 1 | 1 | 9 |
| Girometti (2006) [[14](#_ENREF_142)0] | 1 | 0 | 1 | 0 | 1 | 1 | 1 | 1 | 9 |
| Huang (2005) [[14](#_ENREF_143)1] | 1 | 0 | 0 | 0 | 1 | 1 | 0 | 1 | 9 |
| Kaneko (1994) [[14](#_ENREF_144)2] | 1 | 0 | 1 | 0 | 0 | 0 | 0 | 0 | 9 |
| Kim (2010) [[14](#_ENREF_145)3] | 1 | 0 | 0 | 0 | 1 | 1 | 1 | 1 | 9 |
| Rezasoltani (2010) [[14](#_ENREF_146)4] | 0 | 1 | 1 | 0 | 1 | 1 | 1 | 1 | 9 |
| Rezasoltani (2012) [[14](#_ENREF_147)5] | 0 | 1 | 1 | 0 | 0 | 0 | 1 | 0 | 9 |
| Shinozaki (2003) [[14](#_ENREF_148)6] | 1 | 0 | 1 | 0 | 0 | 0 | 1 | 1 | 9 |
| Sundström (2006) [[14](#_ENREF_149)7] | 0 | 0 | 1 | 0 | 1 | 1 | 0 | 1 | 9 |
| MacGillivray (1998) [[1](#_ENREF_150)48] | 1 | 0 | 0 | 0 | 0 | 0 | 0 | 1 | 8 |
| Tracy (2010) [[1](#_ENREF_151)49] | 0 | 0 | 1 | 0 | 0 | 0 | 1 | 1 | 8 |
| Vecchio (1992) [[15](#_ENREF_152)0] | 0 | 0 | 0 | 0 | 0 | 0 | 0 | 1 | 8 |
| Harrison (2011) [[15](#_ENREF_153)1] | 0 | 0 | 0 | 0 | 1 | 1 | 1 | 1 | 7 |
| Ko (2006) [[15](#_ENREF_154)2] | 1 | 0 | 1 | 0 | 0 | 0 | 0 | 0 | 7 |
| Larsson (1990) [[15](#_ENREF_155)3] | 0 | 0 | 0 | 0 | 1 | 1 | 0 | 1 | 7 |
| McGinley (2012) [[15](#_ENREF_156)4] | 0 | 0 | 1 | 0 | 0 | 0 | 1 | 1 | 7 |
| Wallny (1999) [[15](#_ENREF_157)5] | 0 | 1 | 0 | 0 | 0 | 0 | 1 | 0 | 7 |
| Leistad (2008) [[15](#_ENREF_158)6] | 1 | 0 | 1 | 0 | 0 | 0 | 1 | 1 | 6 |
| Di Mario (2005) [[15](#_ENREF_159)7] | 0 | 0 | 1 | 0 | 0 | 0 | 0 | 0 | 4 |
| Mani (1989) [[1](#_ENREF_160)58] | 0 | 0 | 0 | 0 | 0 | 0 | 0 | 0 | 4 |
| Breidahl (1998) [[1](#_ENREF_161)59] | 0 | 0 | 0 | 0 | 0 | 0 | 0 | 0 | 3 |
|  |  |  |  |  |  |  |  |  |  |
| Percentage of ”yes” scores | 51 | 53 | 80 | 10 | 84 | 74 | 83 | 89 |  |
