# Supplementary material for: Systematic review of quantitative imaging biomarkers for neck and shoulder musculoskeletal disorders
Source: BMC Musculoskelet Disord. 2017 Sep 12;18:395. doi: 10.1186/s12891-017-1694-y (PMC5596923; doi:10.1186/s12891-017-1694-y)
Supplement: Supplementary file 7 — Overview with descriptive information of included studies, by anatomical region of the disorder. (DOCX 112 kb) [file 12891_2017_1694_MOESM7_ESM.docx]

| Additional file 7. Overview with descriptive information of included studies, by anatomical region of the disorder. | | | | | | | |
| --- | --- | --- | --- | --- | --- | --- | --- |
| **MSD Classification or diagnosis**  **Author(s)** | **Biomarker** | **Imaging modality & anatomical location** | **Study population*;**  **Sample size (n)** | **Mean age in years**  **(range if available)** | **Gender** | **Study design** | **Experimental condition** |
| **Neck disorders & symptoms** | | | | | | | |
| Neck pain  Dibai Filho (2012) [[37](#_ENREF_37)] | Skin temperature | Infrared thermography- center of trapezius | Cases (n = 18) & controls (n = 18): university community of Piracicaba, São Paulo, Brazil. | Cases: 22.7 (SD = 3.9);  Controls: 21.6 (SD = 3.1) | F | Control | At rest |
| Neck pain  Elliott (2008) [[46](#_ENREF_46)] | Fat index indicating fatty infiltration (relative fat) | MRI - cervical extensor muscles (suboccipitals, multifidus, semispinalis cervicis, semispinalis capitis, splenius capitis, upper trapezius) | Cases (n = 23) & controls (n = 79):  Cases: recruited through advertising in physiotherapy practices & university fraternity. Controls: chronic whiplash-associated disorder patients from prior study - referral from physical therapists and general medical practitioners in Denver and Brisbane metropolitan areas. | Cases: 29.2 (18-45); Controls: 29.7 (18-45) | F | Control | At rest |
| Neck pain  Falla (2004) [[38](#_ENREF_38)] | Subcutaneous thickness | Ultrasound -sternocleidomastoid (SCM) and anterior scalene (AS) | Cases (n = 20) & controls (n = 20): origin of study subjects not stated. | Cases: 29 (18-47); Controls: 30 (21-51) | M/F | Control | At rest |
| Neck pain  Fernández-de-las-Peñas (2008) [[47](#_ENREF_47)] | Multifidus cross sectional area (CSA), muscle shape ratio (LD/APD) | Ultrasound - cervical multifidus at C3, C4, C5, C6 | Cases (n = 20) & controls (n = 20):  Cases: patients referred by their primary physician to a private clinic of physical therapy. Controls: recruited from advertisement in a local newspaper. | Cases: 34 (SD = 5); Controls: 33 (SD = 6) | F | Control | At rest |
| Neck pain  Javanshir (2011) [[48](#_ENREF_48)] | Longus colli CSA, anterior-posterior dimension (APD), lateral dimension (LD), and shape ratio (LD/APD) | Ultrasound - longus colli | Cases (n = 20) & controls (n = 20):  Cases: patients referred to a physical therapy clinic. Controls: origin not stated | Cases: 31 (SD = 5) Controls: 30 (SD = 6) | M/F | Control | At rest |
| Neck pain  Jesus-Moraleida (2011) [[49](#_ENREF_49)] | Longus colli & SCM thickness changes during test & during rest = proportion of muscle recruitment | Ultrasound - longus colli, SCM | Cases (n=31) & controls (n=31): origin of study subjects not stated. | Cases: 29.7 (SD = 8.8); Controls: 29.7 (SD = 9.0) | M/F | Control | Participants instructed to perform a nodding movement, representing craniocervical flexion test (CCFT) in 5 incremental levels of pressure: 22, 24, 26, 28, & 30 mm Hg (phases 1, 2, 3, 4, & 5, respectively). Participants performed action at each level of pressure holding the target pressure for 10 sec, with 30 sec of rest between levels. |
| Neck pain  Karimi (2016) [[53](#_ENREF_53)] | Dorsal neck muscle thickness change w. 50% & 100% shoulder MVC in 6 directions | Ultrasound –  Dorsal neck muscles (trapezius, semispinalis capitis, semispinalis cervicis, splenius capitis, multifidus) | Cases (n = 17) & controls (n = 20);  All: participants of unknown origin (paper states convenience sample) | Cases: 29 (19-35)  Controls: 27 (19-35) | M | Control | At rest, 50% & 100% shoulder MVC in 6 directions (flexion, extension, external & internal rotation, abduction, adduction) |
| Neck pain  Park (2013) [[50](#_ENREF_50)] | Mean difference in bilateral semispinalis capitis muscle thickness | Ultrasound - semispinalis capitis muscle | Cases (n = 20) & controls (n = 20): graduate students of Yonsei University, Korea. | Cases: 23.4 (SD = 1.9) Controls: 23.4 (SD = 2.1) | M/F | Control | At rest |
| Neck Pain  Rahnama (2015) [[52](#_ENREF_52)] | Multifidus muscle thickness change w. 100% shoulder MVC in 6 directions | Ultrasound – multifidus muscle | Cases (n = 20) & controls (n = 20);  workers recruited from bank office | Cases: 28.9 (SD = 5.5) Controls: 27.5 (SD = 4.4) | M | Control | At rest, 100% shoulder MVC in 6 directions (flexion, extension, external & internal rotation, abduction, adduction) |
| Neck pain  Sheard  (2012) [[51](#_ENREF_51)] | Differences in water relaxation values (T2 relaxation) quantified from scans before & after exercise were calculated (T2 shift) as measure of SA muscle activity. | MRI - 4 intervertebral levels: T6-7, T7-8, T8-9, and T9-T10 | Cases (n=10) & controls (n=10): recruited through advertisements in local and university press. | Cases: 28.2 (SD = 5.3); Controls: 24.9 (SD = 3.2) | M/F | Control | Baseline MRI; 20% MVC isometric shoulder elevation (1 min); rest (30 sec ); 20% MVC isometric shoulder limb elevation (1 min); rest (30 sec ); 20% MVC isometric shoulder elevation (1 min ); rest post-exercise MRI (30 sec ). |

**Shoulder disorders & symptoms**

| Degenerative rotator cuff lesion  Biberthaler (2003) [[54](#_ENREF_54)] | Functional capillary density, capillary diameter | Orthogonal polarization spectral imaging - supraspinatus tendon | Cases (n = 11): patients who presented at a shoulder outpatient clinic with clinical signs typical of degenerative rotator cuff lesion. | Cases: 56 (SD = 9) | M/F | Lesion vs. control tissue in tendon | | During surgery |
| --- | --- | --- | --- | --- | --- | --- | --- | --- |
| Rotator cuff tear (full thickness)  Chang (2014) [[56](#_ENREF_56)] | Biceps long tendon (BLT) width, thickness, flattening ratio (width/thickness), cross-sectional area, echogenicity ratio (mean pixel density BLT/ref. deltiod muscle region) | Ultrasound –  shoulder, biceps tendon | Cases (n = 145) & controls (n = 145): patients referred to musculoskeletal ultrasound lab for shoulder joint exam | Cases: 67.3 (SD = 10.4);  Controls: 67.5 (SD = 10.7) | M/F | Control | | At rest |
| Rotator cuff tear  Choo (2014) [[57](#_ENREF_57)] | Rotator cable thickness, width | MR arthography – shoulder | Cases (n = 32) partial-thickness tears,  (n = 55) full-thickness tears & controls  (n = 27): All: patients with shoulder discomfort who underwent an indirect shoulder arthrography procedure. | Partial-thickness tear: 58.4 (49-71)  Full-thickness tear:  60.5 (44-75)  Controls: 44.9 (20-63) | M/F | Control | | At rest (15 min exercise prior to imaging) |
| Rotator cuff tear  Funakoshi (2010) [[55](#_ENREF_55)] | vascularity in 4 ROIs: articular & bursal sides of supraspinatus tendon, medial & lateral sides of bursa | Contrast-enhanced ultrasound,  bilateral - supraspinatus tendon & bursa | Cases (n = 15) & controls (n = 10):  Cases: patients with unilateral rotator cuff tears. Controls: volunteers of unclear origin. | Cases: 65.7 (SD = 11.5) Controls: 30.2 (SD = 3.9) | M/F | Control | | At rest |
| Rotator cuff tear  Hirano (2006) [[39](#_ENREF_39)] | Full vs. partial rotator cuff tear, rotator cuff tear length, amount of subacrominal-subdeltoid bursal fluid | MR arthrography - shoulder | Cases (n = 23) & controls (n = 15):  Cases: patients who underwent an indirect shoulder arthography procedure. Controls: patients with a shoulder MRI. | Cases: 61 (37-76) Controls: 60 (45-71) | M/F | Control with asymptomatic rotator cuff tears | | At rest |
| Rotator cuff tear  Karthikeyan (2015) [[58](#_ENREF_58)] | Total blood flow in 4 supraspinatus zones, in anteromedial zone, in postero-medial zone | Laser Doppler flowmetry –  Supraspinatus tendon | Full-thickness tear (n = 30);  Partial-thickness tear (n = 30);  Controls (n = 30).  All: candidates for arthroscopic shoulder surgery.  Cases: patients with shoulder pain. | Full-thickness tear: 63 (45-88)  Partial-thickness tear: 57 (26-80)  Controls: 30 (18-48) | M/F | Control | | At rest |
| Rotator cuff tear (full-thickness)  Keener (2015) [[35](#_ENREF_35)] | baseline rotator cuff tear width; Width enlargement (defined as ≥ 5 mm compared with that at baseline) percentage | Ultrasound –  shoulder | Rotator cuff tear with anterior supraspinatus cable disruption: (n = 43);  Rotator cuff tear with anterior supraspinatus cable intact: (n = 96).  All: patients with shoulder pain with asymptomatic rotator cuff tear in contralateral shoulder. | Rotator cuff tear with anterior supraspinatus cable disruption: 64.3 (SD = 7.9)  Rotator cuff tear with anterior supraspinatus cable intact: 62.8 (SD = 9.1) | M/F^b^ | Longitudinal | | At rest |
| Rotator cuff tear  Mall  (2010) [[33](#_ENREF_33)] | Rotator cuff tear length, width, & area; Substantial tear progression (transformation of a partial-thickness tear into a full-thickness tear or size increase >5 mm in either width or length of a full thickness tear, compared with that at time of enrollment) | Ultrasound –  shoulder | Cases (n = 195); 44 developed pain in asymptomatic shoulder; 55 did not develop pain in asymptomatic shoulder (others were enrolled < 2 yrs, withdrew, or had inadequate data). All: patients who presented for bilateral shoulder ultrasonography to investigate unilateral shoulder pain, and who were found to have an asymptomatic rotator cuff tear in the contralateral shoulder. | Developed symptoms: 63.3 (SD = 11)  Did not develop symptoms: 63.1 (SD = 9)  Mean follow-up time = 1.93 (SD = 1.2) yrs | M/F | Longitudinal | | At rest |
| Rotator cuff tear (partial & full) or rotator cuff disease  Keener (2015) [[34](#_ENREF_34)] | Rotator cuff tear enlargement (see paper for definition), new shoulder pain | Ultrasound - shoulder | Full thickness tear (n = 118);  partial tear (n = 56).  Controls: (n = 50).  All: patients from clinical practices of 3 surgeons w. pain w. rotator cuff disease in contralateral shoulder.  Cases: asymptomatic rotator cuff tear in one shoulder.  Controls: no rotator cuff tear in one shoulder. | Full thickness tear: 63. 8 (SD = 9);  Partial thickness tear: 59.4 (SD = 10); Controls: 60.7 (SD = 10)  Median follow-up time = 5.1 yrs. | M/F | Longitudinal control (no evidence of tear in asymptomatic shoulder) | | At rest |
| Rotator cuff tear  Moosmayer (2013) [[36](#_ENREF_36)] | Rotator cuff tear size in antero-posterior plane, in mediolateral plane; tear size increase in antero-posterior plane, in mediolateral plane. | Ultrasound – shoulder | Asymptomatic full-thickness tear (n = 50).  18/50 became symptomatic at 3-year follow-up, 32/50 remained asymptomatic.  (n = 30) patients managed for minor orthopaedic conditions unrelated to the shoulder, (n = 20) outpatients in orthopaedic clinic for shoulder condition | 69 (SD = 7.9) | M/F | Longitudinal | | At rest |
| Rotator cuff tear  Terabayashi (2014) [[59](#_ENREF_59)] | Difference in blood flow peak systolic velocity (PSV), resistance index (RI) between sides | Doppler ultrasound – anterior humeral circumflex artery (AHCA), brachial artery (BA) | Rotator cuff tear with night pain (n = 34);  Rotator cuff tear without night pain (n = 13); Controls (n = 20)  Cases: outpatient clinic patients reporting shoulder pain.  Controls: unspecified origin | Rotator cuff tear with night pain: 68.3 (51-86);  Rotator cuff tear without night pain: 65.8 (51-87);  Controls: 64.3 (38-88) | M/F | Control | | At rest |
| Supraspinatus tendinopathy  Arend (2014) [[63](#_ENREF_63)] | Maximal supraspinatus tendon thickness (MSTT) | Ultrasound –  Supraspinatus tendon | Cases (n = 164) & controls (n = 42).  Cases: patients referred for shoulder MRI.  Controls: unclear origin. | Cases: 42 (19-81)  Controls: 39 (18-63) | M/F | Control | | At rest |
| Rotator cuff tendinitis  Cay (2012) [[60](#_ENREF_60)] | Subacromial distance (distance btwn humeral head & acromion) Glenoid APD, humeral head & glenoid articular surface diameters | MRI -  shoulder | Cases (n = 62[69 exams]) & controls (n = 59 [60 exams]).  All: patients with shoulder MRIs | Cases: 48.7 (25-73) Controls: 37.3 (14-61) | M/F | Control | | At rest |
| Rotator cuff tendinosis  Choo (2014) [[57](#_ENREF_57)] | Rotator cable thickness, width | MR arthography – shoulder | Cases (n = 47) & controls (n = 27):  All: patients with shoulder discomfort who underwent an indirect shoulder arthrography procedure. | Cases: 53.3 (20-73)  Controls: 44.9 (20-63) | M/F | Control | | At rest (15 min exercise prior to imaging) |
| Rotator cuff tendinitis  Rechardt (2010) [[61](#_ENREF_61)] | Carotid artery intima-media thickness | Ultrasound - carotid artery | Cases: males: n = 80 (of 2850); females: n = 95 (of 3387). All: population aged >30 years in Finland contacted via Health 2000 survey. Current sample obtained through systematic sampling from health center districts. | Males: 50.8 (95% CI: 50.2-51.3)  Females: 52.9 (95% CI: 52.4-53.4) | M/F | Population based; Control | | At rest |
| Shoulder tendinopathy  Joensen (2009) [[62](#_ENREF_62)] | Supraspina-tus tendon thickness | Ultrasound - shoulder | Cases (n = 64) | Cases: 47.5 (SD = 11.5) | M/F | unaffected side used as control | | At rest |
| Frozen shoulder  (Adhesive  capsulitis)  Li (2011) [[64](#_ENREF_64)] | CHL thickness | MRI -  Glenohumeral joint | Cases (n=72; 72 shoulder joints) & controls (n=60; 120 shoulder joints). Cases: prospectively referred by several upper limb orthopedic surgeons for MR imaging. Controls: individuals with normal shoulder joints also referred for MR imaging. | Cases: 53.5 (33-72) Controls: 50.5 (no range given) | M/F | Control | | At rest |
| Frozen shoulder (Adhesive capsulitis)  Michelin (2013) [[67](#_ENREF_67)] | Joint capsule thickness | Ultrasound-  Shoulder | Cases (n = 20) (unilateral frozen shoulder)  Patients referred for shoulder ultrasound | Cases: 54.8 (42-76) | M/F | unaffected side used as control | | At rest |
| Frozen shoulder  (Adhesive  capsulitis)  Song (2011) [[65](#_ENREF_65)] | Joint capsule thickness in axillary recess, enhancing portion of axillary recess thickness, rotator interval thickness | MR arthography -humerus | Cases (n = 35) & controls (n = 45).  All: patients who underwent MR arthrography | Cases: 50.1  Controls: 48.9 | M/F | Control | | At rest |
| Frozen shoulder  (Adhesive  capsulitis  Zhao (2012) [[66](#_ENREF_66)] | CHL thickness, articular capsule thickness | MRI - shoulder | Cases (n = 60) & controls (n = 60). Cases: patients seen by shoulder surgeons Controls: unstated origin. | Cases: 50.2 (36-74) Controls: 46.9 (no range given) | M/F | Control | | At rest |
| Shoulder impingement syndrome  Daghir (2011) [71] | Subacomial-subdeltoid bursal thickness (various views) | Ultrasound – shoulder | Cases (n = 23) & controls (n = 24). Cases: patients diagnosed with shoulder impingement syndrome & referred for ultrasound. Controls: hospital staff. | Cases: 52.0 (no range given)  Controls: 49.0 (no range given) | M/F | Control | | At rest, and following shoulder abduction |
| Shoulder impingement syndrome  Hébert (2003) [[68](#_ENREF_68)] | AHD | MRI - shoulder | Cases (n = 29) & controls (n = 10). Cases: diagnosed by an experienced orthopedic surgeon. Controls: unclear origin | Cases: 44.3 (SD = 9.2) yrs ^a^  Controls: 34.4 (SD = 8.4) yrs | M/F | Lesion vs. control tissue in tendon | | Subjects moved and actively maintained for 8 sec his/her arm at successive arm elevation positions in flexion at 50°, 70°, 90°, 110° & 130°, and in abduction at 70°, 80°, 90° & 110°. Each series of movements was repeated 2x at each position, 3 consecutive images were acquired. |
| Shoulder impingement syndrome  Karthikeyan (2015) [[58](#_ENREF_58)] | Blood flow in 4 supraspinatus zones | Laser Doppler flowmetry –  Supraspinatus tendon | Cases (n = 30) & controls (n = 30).  All: candidates for arthroscopic shoulder surgery.  Cases: patients with shoulder pain. | Cases: 55 (35-78)  Controls: 30 (18-48) | M/F | Control | | At rest |
| Shoulder impingement syndrome  Leong (2012) [[69](#_ENREF_69)] | AHD, supraspinatus tendon thickness | Ultrasound -  shoulder | Cases (n = 9).  Controls: volleyball players (n = 15), non-overhead sports (n = 13).  All: university students. | Cases: 21.8 (SD = 1.2). Controls: volleyball players: 21.7 (SD = 1.6) & Controls non-overhead sports: 20.8 (SD = 1.3) | M/F | Control | | At rest |
| Shoulder impingement syndrome  Park (2007) [[70](#_ENREF_70)] | Difference in mean skin temperature btwn sides | Thermography -  shoulder (5 regions) | Cases (n = 100) & controls (n = 30): Cases: patients from a shoulder and elbow joint clinic in the Department of Orthopaedic Surgery. Controls: unclear origin | Cases: 52.0 (SD = 11.2) Controls: not stated | M/F | Control | | At rest |
| Shoulder pain w. rotator cuff disease (multiple diagnoses)  Kalra (2010) [[40](#_ENREF_40)] | AHD | Ultrasound - shoulder (acromion & humerus) | Cases (n = 31) & controls (n = 29).  All: recruited through physician offices at University Health System and with flyers posted at Virginia Commonwealth University Health System and Virginia Commonwealth University campus. | Cases: 53.5 (20.0-80.0) Controls: 31.9 (23.0-60.0) | M/F | Control | | Subjects tested in 3 sitting postures: (1) normal resting posture, (2) slouched posture, and (3) upright posture with scapular retraction. In all postures, ultrasound images were obtained at 2 arm angles: at rest with arm at side and in 45° actively maintained coronal plane shoulder abduction. Sequence of postural alterations was counterbalanced by changing posture sequence, but resting arm position was always tested prior to 45° angle. |
| Shoulder pain  O’Sullivan (2012) [[41](#_ENREF_41)] | Muscle thickness | Ultrasound - trapezius (3 regions) | Cases (n = 18) & controls (n = 18).  All: university staff and student population and physiotherapy clinics local to area who responded to advertising. | Cases: 28.3 (SD = 8.4) Controls: 29.3 (SD = 6.6) | M/F | Control | | Trapezius thickness measurements for upper (C5); middle (T1) and lower (T5 & T8) trapezius muscle were collected under 5 conditions, in both resting and contracted states. 0 degrees – rest; 90 degrees sh abduction - rest & contraction; 120 degrees sh abduction – rest & contraction. Order is not specified in the text. |
|  |  |  |  |  |  |  | |  |
| Shoulder pain  Rechardt (2010) [[61](#_ENREF_61)] | Carotid artery intima-media thickness | Ultrasound - carotid artery | Cases: males: n = 257 unilateral pain, n = 171 bilateral pain (of 2850); females: n = 339 unilateral pain, n = 237 bilateral pain (of 3387). All: population aged >30 years in Finland contacted via Health 2000 survey. Current sample obtained through systematic sampling from health center districts. | Males: 50.8 (95% CI: 50.2-51.3)  Females: 52.9 (95% CI: 52.4-53.4) | M/F | Population based; Control | | At rest |
| Shoulder pain (internal impingement pain)  Tuite (2007) [[72](#_ENREF_71)] | Labral length, thick-capsule labrum length, posterior recess angle MR arthrogram | MR arthrography - shoulder | Cases (n = 26) & controls (n = 26). Cases: patients who were overhead-throwing athletes. Controls: patients who were not overhead-throwing athletes. | Cases: 23 (16-38) Controls: 24 (16-39) | M/F | Control | | At rest |
| **NECK/SHOULDER DISORDERS AND SYMPTOMS** | | | | | | | | |
| Neck/shoulder pain  Hallman (2011) [80] | Muscle blood flow (MBF) | Photoplethysmography -trapezius | Cases (n = 23) & controls (n = 22).  All: recruited through public advertising | Cases: 40.5 (SD = 7.1) controls: 40.8 (SD = 7) | M/F | Control | Baseline rest - 15 min with 5 min rest between tests. Cold pressor test (CPT) & static hand grip test (HGT) counter-balanced; deep breathing test always final test. NOTE: MBF not assessed during/after this test. MBF measurements from last 5 min baseline rest & 60 sec segments during and after CPT & HGT. | |
| Neck/shoulder pain  Nilsen (2007) [[42](#_ENREF_42)] | Blood flow | Laser Doppler flowmetry - thumb (bilateral) | Cases: n = 29 & controls: n = 35: Cases: patients mainly referred from primary care centers and physiotherapists. Controls: recruited from public institutions and private companies. | Cases : 48.3 (32-63)  Controls: 41.4 (19-59) | F | Control | Baseline (10 min); mental stress (60 min); recovery (30 min). | |
| Neck/shoulder pain  Shiro (2012) [[8](#_ENREF_80)1] | ΔOHb, ΔHHb, ΔTHb (all change from baseline) | NIRS - trapezius | Cases (n = 12) & controls (n = 14).  All: unclear origin | Cases: 28.7 (SD = 4.6) controls: 29.5 (SD = 4.1) | F | Control | Rest - 15 min exercise - 1 min rest (Relax 1) - 2 min exercise - 1 min rest (Relax2) - 2 min exercise - 1 min rest (Relax3) - 2 min recovery - 5 min | |
| Neck/shoulder pain  Strøm (2009) [[43](#_ENREF_43)] | Blood flow | Laser Doppler flowmetry - bilateral upper trapezius | Cases (n = 24) & controls (n = 28).  All: recruited through advertisements in local papers and the Internet. | Cases: 39 (SD = 6) controls: 33 (SD = 6) | M/F | Control | Instrumentation applied rest - baseline (3 min) computer work task (90 min) recovery (30 min) | |
| Neck/shoulder pain  Takiguchi (2010) [[7](#_ENREF_78)9] | minimal & maximal standardized uptake values (SUV) of [18F]fluorodeoxyglucose (18F-FDG) | PET/CT – trapezius (gluteus maximus used as a control) | Cases (n = 39) & controls (n = 101) | All: 56.0 (SD = 10.2) | M/F | Control | At rest | |
| Cervicobrachial pain syndrome  Larsson (1998) [[11](#_ENREF_116)4] | Microcirculation | Laser Doppler flowmetry - bilateral trapezius | Cases (n = 71) & controls (n = 20).  Cases: 41 with unilateral pain, 30 with bilateral pain that were hospital patients referred for in-patient medical exams. Controls: hospital workers with ordinary, variable tasks, none causing monotonous load to shoulders. | Cases: 44.5 (no range given)  Controls: 44 (25-63) | M/F | Control | Stepwise increased static load for periods of 1 min @ with 1 min of rest between. A) No load - patient was sitting upright in a standard office chair with relaxed, hanging arms (rest position). On command, patient raised straight arms symmetrically in scapular plane (approximately midway btwn abduction and flexion) to subsequently 30°, 60°, 90° & 135°, i.e., the load positions. B) similar to above but with a 1 kg (women) or 2 kg (men) load carried in each hand. C) A fatigue test (up to 10 min) was performed with straight arms elevated at 45° holding a 1 kg (women) or 2 kg (men) load in each hand. Recovery was achieved with hanging arms and no hand load. | |
| Trapezius myalgia  Acero (1999) [[7](#_ENREF_73)4] | Relative blood volume | NIRS - upper trapezius | Cases (n = 9) & controls (n = 9).  All: male students and staff of a Dental School | Cases: 23.2 (SD = 1.7)  Controls: 24.6 (SD = 2.2) | M | Control | Random order of 2 tests: In the cold pressor trial, left foot and ankle were immersed in 4°C cold water for 2 min, while in mock trial, left foot and ankle were put in same container (now without water) for 2 min. After first trial, participants rested for 20 min, with left foot and ankle placed in a 37°C waterbath for first 5 min after end of the cold pressor trial. Relative blood volume continuously recorded for 1 min before, 2 min during, and 5 min after withdrawal of cold stimulation. | |
| Trapezius myalgia  Andersen (2010) [[44](#_ENREF_44)] | Muscle oxygenation (from baseline: change in oxy-hemoglobin, change in de-oxygenated hemoglobin, total hemoglobin) | NIRS - trapezius | Cases (n = 17) & controls (n = 8),  All: employees (age 30–60 years) at 7 companies (2 banks, 2 post office work places, 2 different national administrative offices and 1 industrial production unit). | Cases: 45.2 (SD = 6.2) Controls: 45.1 (SD = 8.5) | F | Control | 20 min submaximal exercise on a cycle ergometer (55% of maximal oxygen uptake (VO2max) for 10 min and was increased to 70% for further 10 min). | |
| Trapezius myalgia  Cagnie (2012) [[7](#_ENREF_74)5] | Oxygen saturation, blood flow | NIRS/laser Doppler flowmetry - bilateral trapezius, upper, middle, and lower (6 pts total) | Cases (n = 10) & controls (n = 10).  All: office workers, performing at least 4 h of computer work as part of daily job duties. | Cases: 38.89 (SD = 10.25)  Controls: 31.22 (SD = 9.05) | Gender not specified | Control | 60 min duration- combination of typing and mousing tasks, including copy-typing and editing text from a typing training program. Measurements every 10 min, starting before computer task. | |
| Trapezius myalgia  Flodgren (2010) [[7](#_ENREF_75)6] | Muscle oxygenation | NIRS - upper trapezius | Cases (n = 14) | Cases: 40 (SD = 8) | F |  | Rest - after inserting MDy probe (90 min),  Rest – baseline (30 min),  Repetitive, physical work (30 min),  Recovery (60 min) | |
| Trapezius myalgia  Peolsson (2008) [[45](#_ENREF_45)] | Strain rate, strain rate RMS - before provocation, after provocation, difference after - before. | Ultrasound with tissue velocity imaging (TVI) - trapezius | Cases (n = 14) & controls (n = 13).  Cases: female out-patients who had been referred to a hospital Pain and Rehabilitation Centre.  Controls: staff and students at the same hospital. | Cases: 38 (24-48) Controls: 43 (36-55) | F | Control | Baseline - 2 arm abductions pain/exercise provocation - cases: repetitive arm abduction until VAS = 6; controls - 6 repetitive arm abductions post-pain/exercise provocation - 2 arm abductions | |
| Trapezius myalgia  Sjøgaard (2010) [[7](#_ENREF_76)7] | Muscle oxygenation (ΔOHb, ΔHHb, ΔTHb) | NIRS - trapezius | Cases (n = 43) & controls (n = 19).  All: recruited from 7 workplaces (blue + white collar), or via advertisements in local newspapers. | Cases: 44 (SD = 9.8)  Controls: 44 (SD = 9.1) | F | Control | Rest after inserting microdialysis probe (120 min),  Rest – baseline (40 min),  Repetitive, low force task (pegboard) (120 min),  Rest – intermediate (10 min),  Mental stress task (Stroop) (30 min),  Recovery (30 min) | |

^a^: This is of total eligible SIS cases (n = 41), including bilateral cases not used in analysis.

^b^: information from Keener (2015) [34].
